# Aβ42 oligomer-specific antibody ALZ-201 reduces the neurotoxicity of Alzheimer’s disease brain extracts

**DOI:** 10.1186/s13195-022-01141-1

**Published:** 2022-12-29

**Authors:** Anders Sandberg, Ernesto Berenjeno-Correa, Rosa Crespo Rodriguez, Michael Axenhus, Sophia Schedin Weiss, Kevin Batenburg, Jeroen J. M. Hoozemans, Lars O. Tjernberg, Wiep Scheper

**Affiliations:** 1 Alzinova AB, Pepparedsleden 1, SE-431 83, Mölndal, Sweden; 2 Department of Human Genetics, Amsterdam UMC location Vrije Universiteit, Amsterdam, Netherlands; 3 Department of Functional Genomics, Vrije Universiteit Amsterdam, Amsterdam, Netherlands; 4 Department of Neurochemistry, Amsterdam UMC location Vrije Universiteit, Amsterdam, Netherlands; 5 Department of Neurobiology, Care Sciences and Society, Karolinska Institutet, Stockholm, Sweden; 6 Department of Neuropathology, Amsterdam UMC location Vrije Universiteit, Amsterdam, Netherlands

**Keywords:** Aβ42-oligomer-specific antibody, ALZ-201, Alzheimer’s disease, Aβ42 oligomers, Aβ42 neurotoxicity, AβCC peptide™ technology

## Abstract

**Background:**

In Alzheimer’s disease (AD), amyloid-β 1–42 (Aβ42) neurotoxicity stems mostly from its soluble oligomeric aggregates. Studies of such aggregates have been hampered by the lack of oligomer-specific research tools and their intrinsic instability and heterogeneity. Here, we developed a monoclonal antibody with a unique oligomer-specific binding profile (ALZ-201) using oligomer-stabilising technology. Subsequently, we assessed the etiological relevance of the Aβ targeted by ALZ-201 on physiologically derived, toxic Aβ using extracts from post-mortem brains of AD patients and controls in primary mouse neuron cultures.

**Methods:**

Mice were immunised with stable oligomers derived from the Aβ42 peptide with A21C/A30C mutations (AβCC), and ALZ-201 was developed using hybridoma technology. Specificity for the oligomeric form of the Aβ42CC antigen and Aβ42 was confirmed using ELISA, and non-reactivity against plaques by immunohistochemistry (IHC). The antibody’s potential for cross-protective activity against pathological Aβ was evaluated in brain tissue samples from 10 individuals confirmed as AD (*n*=7) and non-AD (*n*=3) with IHC staining for Aβ and phosphorylated tau (p-Tau) aggregates. Brain extracts were prepared and immunodepleted using the positive control 4G8 antibody, ALZ-201 or an isotype control to ALZ-201. Fractions were biochemically characterised, and toxicity assays were performed in primary mouse neuronal cultures using automated high-content microscopy.

**Results:**

AD brain extracts proved to be more toxic than controls as demonstrated by neuronal loss and morphological determinants (e.g. synapse density and measures of neurite complexity). Immunodepletion using 4G8 reduced Aβ levels in both AD and control samples compared to ALZ-201 or the isotype control, which showed no significant difference. Importantly, despite the differential effect on the total Aβ content, the neuroprotective effects of 4G8 and ALZ-201 immunodepletion were similar, whereas the isotype control showed no effect.

**Conclusions:**

ALZ-201 depletes a toxic species in post-mortem AD brain extracts causing a positive physiological and protective impact on the integrity and morphology of mouse neurons. Its unique specificity indicates that a low-abundant, soluble Aβ42 oligomer may account for much of the neurotoxicity in AD. This critical attribute identifies the potential of ALZ-201 as a novel drug candidate for achieving a true, clinical therapeutic effect in AD.

**Supplementary Information:**

The online version contains supplementary material available at 10.1186/s13195-022-01141-1.

## Background

The metabolism of amyloid precursor protein (APP; Entrez gene ID: 351) into aggregation-prone amyloid-β (Aβ) peptides is proposed to be an early upstream factor in the pathogenesis of Alzheimer’s disease (AD) [1, 2]. This notion is supported by genetic evidence from detrimental [3–6] and protective [7, 8] mutations in APP, and alterations in Aβ levels are instrumental for early diagnosis of AD [9]. Multiple factors contribute to the pathological cascade in AD and, while mechanistic connections are still largely elusive [10], a central role is attributed to Aβ42 aggregation in the brain [11–13].

The majority of Aβ generated in human brain comprises Aβ_1-40_ (Aβ40) and Aβ_1-42_ (Aβ42) variants, approximately 80–90% and 5–10%, respectively [14]. Aβ42 has a high propensity to form insoluble fibrillar aggregates deposited as plaques and soluble Aβ oligomers (AβOs) [15, 16]. These oligomers are toxic, soluble Aβ42 assemblies found in human AD brain [17–19] or cerebrospinal fluid (CSF) [20, 21] that correlate with neuroinflammation [22–24], plaque load, synaptic loss and cognitive impairment [25–28]. They are also associated with cognitive deficits [12, 21, 29] more so than plaque load [25, 30, 31]. In experimental models of AD, AβOs trigger tau phosphorylation and aggregation, neuroinflammation, synaptic dysfunction, neurodegeneration and cognitive impairment [32–38].

Given the close association of Aβ42 to early AD pathogenesis, there is a rationale for anti-amyloid immunotherapies, especially those that prevent AβO formation and/or increase their clearance [39]. Immunotherapy strategies, which target brain Aβ clearance actively using Aβ antigens or passively using monoclonal antibodies (mAbs), have been evaluated in large-scale clinical trials [40, 41] and their different binding properties and specificities for Aβ polymorphisms have been extensively reviewed [42–44]. Although the FDA approved aducanumab (Aduhelm®) as the first mAb therapy for AD [45], Aβ-targeting strategies have been largely unsuccessful and have yet to demonstrate conclusively a robust impact on cognitive decline [46–48].

The clinical limitations of current anti-Aβ mAbs may be a consequence of both low antibody penetration into the brain [49–51] and extensive target distraction, i.e., off-target binding to non-toxic Aβ in the brain and other tissue. Plasma concentrations of Aβ are in the order of around 60 pM [52] with a half-life rate of approximately 3 h [53], providing a steady stream of potential off-target interactions for circulating mAbs. In AD brain, Aβ concentrations are higher than in plasma. Soluble Aβ levels in homogenised AD brain extracts are reported as being in the 10 nM range, where only a very low percentage of the total mass concentration of all soluble Aβ appears to be oligomeric [54]. Interestingly, non-homogenised brain extracts have diffusible soluble Aβ levels in the 1-nM range with a similar relative proportion of oligomeric species yet they retain their full toxicity to primary neurons [54]. Since oligomers are composed of a plurality of Aβ molecules, the molar concentration of neurotoxic AβO in brain tissue is thus likely to be very low. Furthermore, of all Aβ in AD brains, approximately 97% are insoluble deposits [31]. Non-oligomer-selective mAbs may, therefore, preferentially bind to this abundant pool of inert, non-toxic Aβ in plasma, blood vessels and brain tissue. This target distraction from the relevant neurotoxic species may also damage the already compromised blood-brain barrier (BBB) in AD [55, 56] and increase the risk of cerebral oedema or cerebral microhaemorrhages observed as amyloid-related imaging abnormalities, or ARIAs, in brain magnetic resonance imaging (MRI) [47, 49, 57, 58]. Thus, superior specificity of a mAb for the neurotoxic AβO species in AD may be a prerequisite for achieving a true clinical therapeutic effect with a favourable risk/benefit profile in AD.

Few immunotherapies specifically targeting unique epitopes on pathological, oligomeric Aβ42 species have been successfully developed. Development of such oligomer-specific therapies has been severely hampered by the intrinsic metastability of these aggregates. One way to overcome this issue is to stabilise or structurally constrain the peptide in a conformation believed to be unique to oligomers and use it as immunogen to generate functional antibodies against this structure. One such example is provided by Gibbs et al. [59] who reported the development of PMN310, a mAb directed against a structurally constrained tetrapeptide comprising residues 13 to 16 of the Aβ peptide identified using a computational model of oligomer structure. Another example of rational vaccine design is provided by the AβCC peptide technology that utilises an intramolecular disulphide bond to structurally constrain the full-length Aβ peptide [60]. This technology prevents the conformational switch of toxic soluble AβOs into insoluble fibrils; instead, these peptides are locked in a β-hairpin conformation producing an anti-parallel β-sheet orientation in the aggregates. This has enabled the subsequent development of an extremely stable Aβ42CC oligomer immunogen, which stimulates generation of oligomer-specific antibodies that may offer a novel approach to explore the neurotoxicity of pathological oligomeric Aβ42 species and, potentially, a unique Aβ42 oligomer-specific mAb for therapeutic intervention in AD.

Here, we developed and extensively characterised a murine IgG mAb, denoted ALZ-201, which was generated using Aβ42CC as immunogen. We determined the stability and binding profile of ALZ-201 and demonstrate that it is a high-affinity antibody with specificity for oligomeric Aβ42 and not fibrillar or non-aggregated Aβ42. Importantly, we demonstrate the potential of ALZ-201 to protect against the neurotoxicity of soluble AD brain extracts in cultured primary neurons, providing in vitro proof-of-concept for the efficacy of targeting a low-abundant, toxic Aβ42 species to ameliorate neurodegeneration.

## Methods

### Amyloid-β production and purification

The protocol of Sandberg et al. [60] was followed for all recombinant production of Aβ42 and Aβ42CC used herein, denoted rAβ42 and rAβ42CC, respectively. In brief, the Aβ42 or Aβ42CC peptide was co-expressed with a histidine-tagged Aβ-binding protein in *Escherichia coli*. Cells were lysed and the peptide-protein complex first purified using immobilised metal affinity chromatography on Ni-NTA resin (Sigma) and then purified further using size exclusion chromatography (SEC). The complex was dissociated with 6 M guanidinium chloride and the histidine-tagged Aβ-binding protein was removed by performing immobilised metal affinity chromatography under denaturing conditions, thereby obtaining the Aβ peptide in the flow-through. The Aβ peptide in 6 M guanidinium chloride was concentrated and applied to a SEC column equilibrated with phosphate buffer saline (PBS). Using this protocol, stable oligomeric rAβ42CC is typically obtained by SEC as approximately 100 kDa aggregates in neutral PBS. It was previously demonstrated by circular dichroism that these oligomers contain ~40% β-sheet structure, and transmission electron microscopy (TEM) images revealed them to be spherical oligomers with an average diameter of ∼6 nm [60]. Upon concentration or incubation, they form larger oligomers centred around 700–800 kDa (see Additional Figure 1 in the Supplemental Appendix). These larger structures were previously suggested to be multimers of the 100-kDa oligomer and in TEM images have the appearance of rod-like and slightly curved structures 6 nm in diameter and ranging in length [60] and are, therefore, very similar in size and morphology to the Aβ protofibrils defined as oligomeric structures 6–10 nm in diameter and ranging in length up to 200 nm [61]. Aβ42CC oligomers >100 kDa are known to have the appearance of protofibrils and since there appears to be no clear structural distinction between a 100-kDa Aβ42CC oligomer and an Aβ42CC protofibril except for size, we herein use the term oligomer as an umbrella term for soluble assemblies of Aβ42CC ≥100 kDa.

Monomeric rAβ42 and rAβ42CC were prepared as the oligomeric rAβ42CC derivative bar the following: during the final SEC, the buffer pH was raised to 10.4–10.7 to maintain these aggregation-prone peptides in the unstructured monomeric state. Control measurements indicated that the pH remained stable at 10.4–10.7 throughout the SEC run and during the limited storage period of the obtained solution (kept in a sealed tube on ice or at 4 °C). Preparations of monomeric solutions were used within 24 h. Immediately prior to analysis and/or concentration determination, the pH of the solutions containing eluted monomers was lowered to 7.2–7.4 using HCl.

Synthetic Aβ42CC was custom made by solid-state peptide synthesis and purified with reversed-phase HPLC using standard methods and practices (AmbioPharm Inc., USA). Oligomeric peptide was obtained by dissolving the peptide at pH 10.0–10.4 and then neutralising the solution to initiate oligomerisation.

Attenuated total reflection (ATR) Fourier transform infrared (FTIR) spectroscopy was used to confirm the oligomeric conformation of the Aβ42CC oligomers. This method can accurately distinguish Aβ oligomer from Aβ fibril states based on the different spectral properties of the anti-parallel β-sheet conformation of oligomers and the parallel β-sheet conformation in fibrils [62, 63]. Measurements were performed on a Bruker Tensor 27 FTIR-spectrometer (Bruker Optics GmbH, Germany) using Opus 6.5 software. The instrument was equipped with a Mercury-Cadmium-Telluride detector cooled with liquid nitrogen. Spectra were recorded in the ATR mode using a Golden Gate ATR accessory (Specac, UK) with an integrated total reflection element composed of a single reflection diamond. The angle of incidence was 45°. The sample (1 μL) was loaded onto the crystal and dried with nitrogen flow, and FTIR spectra recorded between 4000 and 600 cm^−1^ at a resolution of 2 cm^−1^. Each spectrum was the average of 128 scans, and five spectra were recorded for each sample. The water vapour contribution was subtracted from the spectra, which were then baseline-corrected and normalised for equal area between 1740 and 1478 cm^−1^.

SEC coupled with multi-angle light scattering (SEC-MALS; 1100 Series from Agilent) was used to analyse the molecular weight distribution of Aβ42CC oligomeric preparations. A 100-μL sample was thawed and injected on a TSK-GEL® G4000SWxl 7.8 X 300 mm column (Tosoh) pre-equilibrated with 20 mM sodium phosphate buffer and 150 mM NaCl, pH 7.4. The auto sampler was held at a temperature of 5 °C to prevent further oligomerisation. The flow rate was 0.6 mL/min. UV at 280 nm and MALS (MiniDAWN Treos from Wyatt Technology Corporation) detection was used to determine the weight average molecular weight (Mw) of each separated sample fraction using ASTRA 6.1 Software (Wyatt Technology).

All concentrations of Aβ42CC and rAβ42CC were determined with a validated UV spectroscopy method using an extinction coefficient of 1401 cm^−1^ M^−1^ for the difference in absorbance at 280 and 300 nm. For rAβ42, an extinction coefficient of 1424 cm^−1^ M^−1^ was used for the same wavelengths, as previously described [64]. For monomeric and fibrillar preparations of synthetic Aβ42, concentration was inferred from the peptide content as determined by elemental analysis by the manufacturer of the peptide (Bachem).

### Monoclonal antibody development

#### Murine ALZ-201

Six BALB/c mice were immunised with rAβ42CC oligomers using complete or incomplete Freund’s adjuvant (CFA and IFA, respectively) according to the following schedule: Day 0: CFA + 100 μg rAβ42CC oligomers; Day 14: 75 μg rAβ42CC oligomers; Day 28: IFA + 75 μg rAβ42CC oligomers; Day 42: 75 μg rAβ42CC oligomers; Day 56: IFA + 75 μg rAβ42CC oligomers. The two mice with the best titres in a direct enzyme-linked immunosorbent assay (ELISA) against rAβ42CC oligomers were selected for fusion. These mice were again administered 100 μg rAβ42CC oligomers and splenectomised 72 h later. Fusion was carried out according to standard procedures using polyethylene glycol and dimethyl sulphoxide [65, 66]. Two and three weeks post fusion, hybridoma supernatants were screened for positive reactivity towards rAβ42CC oligomers and negative reactivity towards rAβ42CC monomers. Selected clones were expanded and sub-cloned by limited dilution, then frozen and stored in liquid nitrogen and/or at −150 °C in a freezer. Clones selected for expansion and purification were thawed and adapted to serum-free medium.

The isotype was determined using IsoQuick™ Strips for Mouse Monoclonal Isotyping (Sigma-Aldrich). The lead mAb, ALZ-201, was purified by Protein A chromatography [67] and stored at −80 or −20 °C.

The binding affinity of ALZ-201 was determined by an inhibition ELISA based on existing methodology [68, 69]. The antibody was subjected to a pre-incubation step with antigen lasting 1 h at room temperature (RT; 21 ± 1 °C) before the solution was transferred to the Maxisorp plate. After 10 min at RT, the solution was removed and added to an identical plate for 10 min. The dissociation constant (*K*_D_) for the affinity of ALZ-201 for oligomers was defined as the concentration of antigen required to inhibit half of the ELISA signal [68, 69]. IGOR software (Wavemetrics) was used to fit a sigmoidal equation to data that were then analysed as f*, defined as the square root of the fraction of saturated antibody to also take bivalency into account [69].

#### Human IgG1 chimeric ALZ-201

Starting with the sequences for the heavy and light chain (HC and LC, respectively) variable regions of the murine ALZ-201 antibody, a chimeric full-length human IgG1 antibody with backbones for human IgG1 HC constant region and human kappa LC constant region was designed. The cDNA sequences were synthesised and sub-cloned into mammalian cell expression vectors and transiently transfected into Chinese Hamster Ovary (CHO) cells (XtenCHO cells; Proteogenix). A 30 mL culture was fermented in medium, and culture medium collected after 14 days when viability dropped below 50%. The antibody was purified on a Protein A resin using standard procedures: (1) clarification by 0.22 μm filtration, (2) equilibration, binding and washing in PBS pH 7.5, (3) elution by pH shift with Tris-Glycine at pH 2.7, (4) neutralisation with Tris-HCl pH 8.5 and (5) buffer exchange to PBS pH 7.5. The purity of the pooled fractions was determined by SDS-PAGE and concentration by UV spectrophotometry at 280 nm using an extinction coefficient of 230,000 M^−1^ cm^−1^. The chimeric ALZ-201 antibody was named chALZ-201.

### Validation of binding specificities of ALZ-201 and chimeric ALZ-201

#### ALZ-201 conformational specificity for oligomeric Aβ42CC

The stability and conformational specificity of ALZ-201 for oligomeric Aβ was validated by assessing its reactivity towards varying degrees of Aβ42CC aggregation. Lyophilised Aβ42CC peptide was solubilised at RT in a high-pH solution (pH 10) and then diluted to 0.6 mg/mL in 20 mM sodium phosphate, 150 mM NaCl. For one fraction, the pH was kept at 10 during coating of the ELISA plates to prevent oligomerisation. For the other fraction, the pH was adjusted to 7.2 to initiate the oligomerisation process. This fraction was passed through a 0.22-μm filter (Millex GV, Millipore) and aliquoted into 1-mL samples. Fifty minutes after the pH neutralisation step, 1/3 of the tubes were removed and transferred to −20 °C storage. The remaining tubes were incubated at RT for either 8 h (1/3 of the tubes) or 24 h (1/3 of the tubes) before transferring to −20 °C storage. Additionally, the state of Aβ42CC oligomerisation of the samples prepared under neutral conditions was confirmed using SEC-MALS.

For the ELISA, all four samples were diluted to 500 ng/mL in 1× PBS pH 7.4, except for the high-pH sample, which was maintained at pH 10, and 96-well microplates (Nunc Maxisorp) were coated with 100 μL solution at 5 ± 3 °C for 16 h. Plates were washed 3× with 300 μL/well of PBS and 0.05% Tween-20, and the plates blocked with 200 μL/well of blocking buffer (1% bovine serum albumin, BSA, in 1× PBS) and incubated for 45 min at 37 °C. After removing the blocking buffer, a concentration series of either the positive control antibody 6E10 (binds monomers, oligomers and fibrils with high affinity) or ALZ-201, both diluted in PBS with 0.1% BSA, was added at 100 μL/well and allowed to bind for 2 h at 21 °C. After washing as above, 100 μL/well of secondary antibody (goat anti-mouse IgG HRP-conjugated; Southern Biotech) at 0.5 μg/mL in PBS with 0.1% BSA was added. Plates were incubated at 21 °C for 2 h and washed as above. One hundred microlitres/well of 1 mg/mL o-phenylenediamine dihydrochloride in 0.05 M phosphate-citrate buffer, pH 5.0 and 0.02% H_2_O_2_ was added, and the 450–630 nm absorbance recorded after approximately 15 min.

Data were analysed by fitting a 4-parameter logistic function (*Y*= (*A−D*)/(1+(*X*/*C*)^*B*)+*D*) to the measured absorbance values and extracting the maximum response (*Y*max) and half maximal effective concentration (EC50).

#### ALZ-201 conformational specificity for oligomeric Aβ42

The effect of ALZ-201 on fibril formation was assessed with Thioflavin-T (ThT) aggregation assays on Aβ42 using rAβ42 and ALZ-201 at concentrations of 15 and 2 μM, respectively. The buffer used was TBS with 10 μM ThT. Aggregation assays were performed using a FLUOStar Optima reader (BMG) equipped with 440-nm excitation and 480-nm emission filters. The assays were carried out at 37 °C with orbital shaking between data points.

A sandwich ELISA was developed to detect and quantitate ALZ-201-positive oligomeric rAβ42. ALZ-201 was biotinylated with the EZ-Link sulfo-NHS-biotin kit (Thermo Scientific) according to the manufacturer’s instructions. Maxisorp plates (Nunc) were coated with 240 ng ALZ-201 at 4 °C for 15 h, washed three times with PBS, 0.05% Tween-20, and were then blocked with 1% BSA in PBS at RT for 1 h. After removal of the blocking solution, plates were treated with antigen (see next paragraph) for 2 h at RT and then washed as described above. Approximately 350 ng biotinylated ALZ-201 in 100 μL PBS with 0.1% BSA was then allowed to equilibrate with captured antigen for 1 h at RT. After washing as described above, the HRP-conjugated Streptavidin (R&D Systems) diluted 1:200 in PBS with 0.1% BSA was added, and the plates were incubated for an additional hour at RT. Washing as above removed unbound HRP-Streptavidin, and bound enzyme was detected spectrophotometrically at 450 nm using 1 mg/mL o-phenylenediamine dihydrochloride substrate in 100 mM Na_3_-citrate-HCl buffer at pH 4.5 with 0.012% H_2_O_2_.

Antigen was prepared as follows: 100 μL samples of monomeric rAβ42 (prepared under high-pH conditions) at 20 μM in 10 mM Tris-HCl, 150 mM NaCl and pH 7.4 were allowed to aggregate in 1.5-mL Eppendorf tubes at 37 °C and orbital shaking at 600 rpm for 0, 5, 10, 15, 20, 25, 30, 35, 40, 45, 50 and 60 min. Triplicates of all samples were prepared, except for time points 35, 40 and 50 min for which *n*=2. This experiment started with the aggregation of the 60-min sample, and all other samples were incubated on ice until it was time to start the aggregation (incubating on ice prevents aggregation). With this protocol, all samples reached the end of their respective aggregation times at the same time, at which point they were allowed to cool on ice for 5 min. They were then diluted with 1% BSA to give a final concentration of 0.1% BSA and were added to the ALZ-201-coated and BSA-blocked Maxisorp plates, and the sandwich ELISA was subsequently carried out as described in the previous section. Standard curves of rAβ42CC oligomers were prepared and analysed in parallel.

#### Validation of antibody binding specificities

Non-reactivity towards fibrillar and monomeric Aβ42 was confirmed for both murine ALZ-201 and chALZ-201 using a direct ELISA protocol. In this experiment, they were benchmarked alongside biosimilar mAbs, specifically the plaque-targeting mAbs lecanemab (ProteoGenix), aducanumab (TAB-707; Creative Biolabs) and gantenerumab (ProteoGenix). All biosimilar mAbs and the chALZ-201 were of the human IgG1 isotype.

The monomeric, unstructured, Aβ42 peptide antigen (H-1368; Bachem) was prepared by reconstitution of lyophilised peptide at 1 mg/mL in 0.1 M aqueous ammonia solution (pH 9). The fibrillar Aβ42 peptide antigen (H-1368; Bachem) was prepared by reconstituting lyophilised peptide at 1.0 mg/mL in PBS with 0.02% azide and shaking the solution for 55 h at 700 rpm and 37 °C, after which the solution was incubated for 90 h without shaking at RT before being frozen at −20 °C. Oligomeric Aβ42CC was prepared by reconstituting lyophilised peptide at 0.6 mg/mL in PBS and allowing it to aggregate at RT to an average molecular weight of 702 ± 3.5 kDa and with an oligomer content of >94% as determined by SEC-MALS (see Additional Figure 2 in the Supplemental Appendix). Oligomers were then frozen to prevent further oligomerisation. Frozen vials of both fibrillar Aβ42 and oligomeric Aβ42CC were thawed immediately before application to ELISA plates, whereas monomeric Aβ42 was prepared fresh and used within 2 h of preparation.

Nunc Maxisorp™ ELISA plates (Invitrogen) were coated with 5 μg/mL antigen (100 μL/well) for 2 h at 37 °C. Plates were then blocked with 1% BSA in PBS (150 μL/well) for 40 min at 37 °C. Washing between steps was carried out using PBS with 0.05% Tween-20 (300 μL/well) three times. All primary mAbs were diluted from 1000 to 0.46 ng/mL with 0.1% BSA in PBS, added at 100 μL/well and incubated for 1.5 h at RT. After washing as described above, a HRP-conjugated secondary mAb was added at 1000 ng/mL (100 μL/well) and the plates incubated for 45 min at 37 °C. Plates were again washed, after which 3,3′,5,5′-tetramethylbenzidine substrate was added (100 μL/well), and the plates were incubated for 5–10 min at 37 °C. The reaction was stopped with 2 M HCl (50 μl per well), and the difference in absorbance at 450 and 630 nm was measured using a spectrophotometer. The data were analysed as described above.

Immunohistochemistry was used to confirm that non-reactivity towards synthetic fibrillar Aβ translates to non-reactivity towards fibrillar deposits, or plaques, in human tissue. Hippocampal tissue sections were obtained from the Netherlands Brain Bank, 4 μm thin, from patients with AD (*n*=3) and non-AD controls (*n*=3). All AD subjects met the criteria for definitive AD according to the Consortium to Establish a Registry for AD (CERAD). The control subjects had no known psychiatric or neurological disorders. The antibodies used were 6E10 (BioLegend), lecanemab biosimilar (Proteogenix) and chALZ-201. Sections were deparaffinised and hydrated, first twice in xylene and then in a decreasing concentration of ethanol using 99.5, 95 and 70% ethanol w/v before being placed in distilled H_2_O (dH_2_O). Each incubating step of the hydration process took 10 min. Sections were autoclaved for antigen retrieval in DIVA decloaker bath, at 110 °C for 10 min before being washed with dH_2_O.

The protocol for IHC using human primary antibodies was adapted from the *Human-on-human IHC* kit from Abcam (ref: ab214749), and the protocol for mouse primary antibody was adapted from the *MACH1 universal HRP polymer detection* technology from BioCare Medical (ref: M1U539) as follows: lecanemab and chALZ-201 were diluted to a concentration of 0.1 μg/ml in human primer and incubated at 4 °C overnight (approximately 16 h). Sections were peroxidase-blocked at RT for 10 min followed by washing with PBS containing 0.05% Tween20 (PBS-T) for 3 × 5 min with gentle rocking. Quenching buffer was added to the human antibody solutions at a ratio of 1:5, which were incubated for 30 min at RT. Blocking buffer A was added to the sections, which were incubated for 30 min at RT, and then washed with PBS-T for 3 × 5 min. Blocking buffer B was then added and the slides were incubated for 5 min at RT. Sections were washed with PBS-T 3 × 5 min before being incubated with the primary human antibody solution for 16 h at 4 °C. Sections were washed with PBS-T 3 × 5 min before incubation with Human HRP polymer for 10 min at RT. 3,3'-Diaminobenzidine (DAB) solution was prepared by adding one drop of DAB Chromogen to 1 ml of DAB substrate buffer. After an additional wash with PBS-T 3 × 5 min, the DAB solution was applied and sections incubated for 5 min at RT. They were then washed with dH_2_O for 2 min before being dehydrated in rising concentrations of ethanol and xylene.

Sections stained for the murine 6E10 were peroxidase-blocked and washed as described above before being blocked using Normal Goat Serum (NGS) for 20 min at RT and incubated for 16 h at 4 °C with 0.1 μg/ml 6E10 in 3% NGS in PBS. After washing with PBS-T 3 × 5 min, sections were incubated with mouse probe for 30 min at RT before being washed again with PBS-T 3 × 5 min. They were then incubated with universal HRP polymer for 30 min at RT and washed with PBS-T 3 × 5 min. DAB solution incubation and section rehydration were performed as described above.

All sections were mounted using a xylene-based mounting medium Vectamount (Thermo Fisher), covered with cover slips with a refractive index of 1.0 and observed using a Nikon Camera DS-Qi2 (Nikon) with capture software Nikon NIS-elements. Colour correction channels were adjusted for whitening, and conditions were kept the same for each image capture. The image capture centre was localised at the hippocampal subregion of CA3, and images were captured using a ×20 objective. Quantification of signal intensity was done using ImageJ imaging software (version 1.44; NIH, UK). A mask was drawn around the CA3 hippocampal subarea and whole regional intensity was measured; data have been displayed in the interval of 0–10 with 10 being highest intensity. Graphs were made in GraphPad (version 9.4.0; GraphPad Software).

### Neuropathological classification of human brain tissue, whole-brain extract preparation and measurement of protein and amyloid-β content

Human brain tissue was obtained as a fresh-frozen tissue block (approximately 5 g of white matter w/w) from the Netherlands Brain Bank. Each tissue sample was used for sectioning for neuropathological confirmation of AD (test) or non-AD (control) followed by preparation of brain extracts. Frozen sections (5 μm) from cortical (temporo-parietal grey matter cortices/frontal cortex) brain tissue samples from a total of 11 individuals were analysed by IHC using the murine primary mAbs with N-terminal a.a. 1–16, 6E10 (Sigma-Aldrich) and AT8 (phospho-PHF-tau pSer202+Thr205; Thermo Fisher Scientific) for Aβ and p-tau, respectively, and the Dako Envision goat anti-mouse/rabbit-HRP K5007 (Agilent Technologies) as a secondary antibody.

Human brain tissue samples were also isolated post mortem from the temporo-parietal grey matter cortices/frontal cortex of the AD and control human subjects, homogenised in artificial cerebrospinal fluid (aCSF) and centrifuged at 22,000*g* for 110 min, and the supernatant was collected for subsequent analysis.

Whole-brain extracts (supernatant) were dialyzed using Slide-Al-Lyzer dialysis cassettes, 2K MWO (Thermo Fisher Scientific), aliquoted, snap-frozen and stored at −80 °C. Total protein content of the brain extracts was measured using the Pierce BCA Protein Kit according to the manufacturer’s protocol (Thermo Fisher Scientific). The Aβ40 and Aβ42 concentrations were determined with the MesoScale Discovery platform using the Amyloid-β Peptide Panel 1 Kit (4G8: Aβ42, Aβ40, Aβ38) according to the manufacturer’s protocol (Meso Scale Diagnostics).

### Immunodepletion of brain extracts

For immunodepletion, a sample of brain extract (500 μl) was incubated with 1 μg mAb overnight at 4 °C under continuous rotation. The following day, 50 μl of 10% Protein G-sepharose beads (Abcam) in PBS were added and the mixture was incubated for 4 h at 4 °C under rotation. Subsequently, samples were centrifuged at 10 000*g* for 10 min at 4 °C to remove the beads. Supernatants were collected, snap-frozen in liquid nitrogen, and stored at −80 °C until use. Brain extracts were immunodepleted with ALZ-201, 4G8 (positive control; BioLegend) or IgG3 (negative isotype control to ALZ-201; GeneTex).

### Isolation and culture of primary mouse neurons

Animal experiments for primary cell culture were in accordance with institutional and Dutch governmental guidelines and regulations and were approved by the Animal Ethical Committee of the VU University/VU University Medical Centre. Embryonic day 18.5 wild-type mouse embryos obtained by Caesarean section of pregnant females from timed mating were used for primary neuronal cultures. Cortices were dissected in Hanks Buffered Salt Solution (HBSS; Sigma-Aldrich) containing 10 mM 4-(2-hydroxyethyl)-1-piperazineethanesulfonic acid (HEPES) buffer (Gibco) (Hanks–HEPES) and digested by addition of 0.25% trypsin (Gibco) for 20 min at 37 °C. Digested tissue was washed three times in Hanks–HEPES and subsequently triturated with fire-polished Pasteur pipettes in Dulbecco’s modified Eagle’s medium (DMEM) containing 4.5 g/L glucose and UltraGlutamine I (Lonza) supplemented with 10% heat-inactivated foetal bovine serum (HI-FBS), 1% penicillin/streptomycin (pen-strep; Fisher Scientific) and 1% non-essential amino acid solution (Fisher Emergo) (DMEM+). Dissociated cells were spun down, re-suspended and plated in Neurobasal culture medium (NB; Fisher Scientific) supplemented with 2% B-27 (Life Technologies), 18 mM HEPES, 0.25% glutamax (Fisher Scientific) and 0.1% pen-strep (NB+). The re-suspended cells were plated in black-based, 96-well plates (Greiner Bio-One BV) at a cell density of 15K per well and cultured at 37 °C and 5% CO_2_.

### Cell treatments and evaluation of protein concentrations

Samples were diluted to a total protein concentration of 40 μg/ml in NB+. Half of the total volume (50 μl) of the NB+ of each well was removed and substituted with 50 μl pre-warmed sample dilution to obtain a final concentration of 20 μg/ml for 24 h in six replicate wells. The different brain fractions were added to cells day in vitro (DIV) 17. Cells were fixed on DIV18 by incubation with 1.85% paraformaldehyde (PFA; Merck Millipore) in PBS at pH 7.4 for 10 min by replacing half the volume of the media with 3.7% PFA in PBS. Subsequently, the medium plus PFA were removed and substituted with 3.7% PFA for 10 min at RT.

### Immunofluorescence and image acquisition and analysis

Fixed cells were permeabilised in 0.5% Triton X-100 (Fisher Emergo) in PBS (pH7.4) for 5 min at RT and blocked in 2% normal goat serum (Gibco) and 0.1% Triton X-100 PBS for 30 min at RT. Neurons were incubated with primary antibodies diluted in blocking solution 16–17 h at 4 °C. The primary antibodies used were vesicular glutamate transporter 1 (vGlut1) rabbit polyclonal (1:1000) (Synaptic Systems), neurofilament H mouse monoclonal (1:1000) (SMI-32P, Eurogentec) and microtubule-associated protein 2 (Map2) chicken polyclonal (1:250) (Abcam). After three 5-min washes with PBS, neurons were incubated with Alexa fluor (546, 647; Life Technologies)-conjugated secondary antibodies (1:500). After one 5-min wash with PBS, nuclei were stained with 4′,6-diamidino-2-phenylindole (DAPI; Brunschwig Chemie bv) diluted in PBS (1:1000). Finally, after two PBS washes, the plates were stored in PBS at 4 °C until microscopic analysis.

High-content automated microscopy was performed on a CellInsight CX7 HCS platform (Thermo Fisher) to obtain 100 images per well. Images were analysed with in-house developed scripts [70] using Columbus 2.5 software (PerkinElmer). Nuclei were detected in the DAPI channel. A Map2-based region of interest (ROI) was used to determine the number of neurite segments, branches (nodes type 1) and extremities. The number of synapses was measured in the vGlut1 channel within the Map2-based ROI. The values of all analysed morphological characteristics were normalised to the number of cell nuclei and represented as percentage change compared to the control condition.

### Statistical analysis

For the ELISA and IHC data, the two-tailed *t* test was used to calculate the significance value (*p* value) of the difference between the observed means in two independent samples, and the 95% confidence interval (CI) of the difference was reported.

For the cell studies, a power analysis was conducted using G*Power 3.1 software that applied the mean and standard deviation per condition. A sample size of *n*=7 AD brains was selected to enable the evaluation of a statistically significant difference per analysed parameter. Statistical analysis and graphing was performed using GraphPad Prism software (Prism 8.4.2, GraphPad Software Inc., EEUU). The Shapiro-Wilk test was used for normality testing and outliers were excluded from analysis using the ROUT method (*Q*=1%). One-way analysis of variance (ANOVA) with Dunn’s or Dunnet’s post hoc test was used for multiple comparisons. The type of data normalisation has been indicated in the figure legend. A *p* value <0.05 was considered statistically significant. For each dataset, the statistical test and exact significance values have been indicated in the figure or legend. All brains analysed were included in the analyses.

## Results

### Monoclonal antibody development and characterisation

#### ALZ-201 and chALZ-201 development

To address the limitations of existing immunotherapies as regards their Aβ binding properties and consequent clinical effects, hybridoma technology was used to create a mAb specific for Aβ42 oligomers and evaluate its binding profile. Using AβCC peptide technology [60], stable rAβ42CC oligomers were administered to BALB/c mice to generate hybridomas producing antibodies reactive towards rAβ42CC oligomers, but non-reactive towards rAβ42CC monomers. Of the stable clones identified, one was selected for expansion and purification and named ALZ-201. The antibody was isotyped as IgG3, and the binding affinity for the antigen (*K*_D_) was measured by an inhibition ELISA experiment to 1.34 ± 0.29 nM (see Additional Figure 3 in the Supplemental Appendix).

Some murine IgG3 antibodies show a tendency towards cooperative binding to multivalent antigens that will result in increased functional affinity [71]. To rule out any such contribution, a chimeric, full-length human IgG1 antibody with backbones for human IgG1 HC constant region and human kappa LC constant region (chALZ-201) was developed by sub-cloning synthesised cDNA sequences into mammalian cell expression vectors and transiently transfecting them into CHO cells. This was also the first step to ALZ-201 humanisation for future development as a potential therapeutic mAb.

Following successful development of both ALZ-201 and chALZ-201, their binding profiles were characterised using ELISA and different aggregated forms of Aβ to determine their unique specificity and affinity.

#### ALZ-201 shows conformational specificity for soluble aggregates of Aβ42CC and affinity for a low-abundant form of Aβ42 oligomers

To evaluate whether ALZ-201 is truly specific and not just selective for structured oligomers, its specificity for soluble aggregates of Aβ42CC was evaluated using an ELISA against increasing levels of aggregated states of Aβ42CC. The antibody 6E10 (BioLegend), directed against a part of the peptide that is unstructured and available for binding in all forms of Aβ used herein (N-terminal amino acids 4–10), was used as a positive control.

**Fig. 1:**
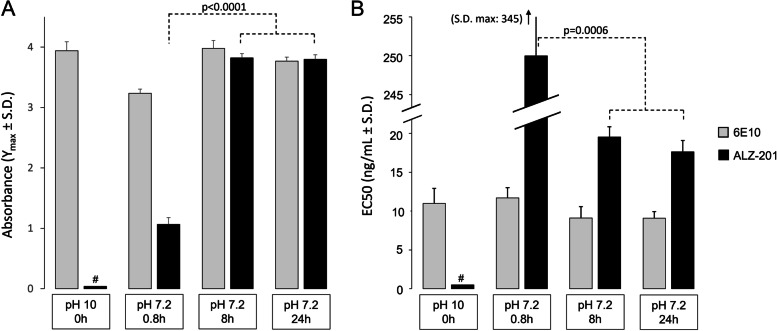
ALZ-201 and 6E10 antibody dose-response curves against increasing levels of Aβ42CC peptide aggregation. The antibody dose-response curves of ALZ-201 and 6E10 against increasing levels of Aβ42CC peptide aggregation are shown, specifically maximum response (*Y*max) (**A**) and half maximal effective concentration (EC50) (**B**). Antibody binding was measured using an optimised and partially validated direct ELISA against immobilised antigen, and the *Y*max and EC50 taken from a 4-parameter logistic equation fitted to the experimental data (see Additional Figure 4 in the Supplemental Appendix for supporting experimental data). After reconstituting the Aβ42CC peptide at high pH, it is non-aggregated and has a random coil structure. Maintaining the peptide at high pH prevents it from aggregating, and this sample was used here as a proxy for a true Time 0 sample that, for technical reasons, is difficult to obtain. #: No binding detected

It is well established that Aβ peptides are largely monomeric under strong alkali conditions [72]. At pH 10, the Aβ42CC peptide is also typically unstructured and monomeric, representing the non-aggregated state. ALZ-201 showed no reactivity towards this form of the peptide. After only 50 min of incubation in neutral pH and PBS without agitation, however, partial oligomerisation of Aβ42CC and a partial signal for ALZ-201 binding was detected (Fig. 1; see also Additional Figure 4 in the Supplemental Appendix). SEC-MALS indicated that in this sample, approximately 20% of the peptides were 104 kDa oligomers, previously shown to be the first β-structured form of the peptide [60], whereas 80% were unstructured monomers or very small oligomers for which the MALS data were too noisy for accurate molecular weight calculations (see Additional Figure 5 in the Supplemental Appendix for SEC-MALS data).

After 8 h of incubation, the Aβ42CC peptide was completely oligomerised and the SEC-MALS data indicated that most of the oligomers (88%) were now about 793 kDa in size, whereas 7% were even larger, eluting in the void with a molecular weight of more than 1 MDa (see Additional Figure 5 in the Supplemental Appendix). The ATR-FTIR spectroscopy showed that these were composed of an anti-parallel orientation of the β-sheet tertiary structure and that the secondary structure content contained 39.8 ± 0.7% β-sheet, 16.5 ± 0.2% turns, 41.1 ± 0.2% coils, but no α-helices (Additional Figure 7 in the Supplemental Appendix). It has previously been shown with both TEM [60] and atom force microscopy [73] that these large oligomers of Aβ42CC are rod-like structures with a smooth curvature that are similar in size and morphology to protofibrils formed by Aβ42 [61]. ALZ-201 showed strong affinity for these oligomers, and the magnitude of the ALZ-201 response was increased by 2.76 absorbance units compared to the partially aggregated sample (1.07 ± 0.11 compared to 3.82 ± 0.07 for the fully oligomerised Aβ42CC; 95% CI, 2.62 to 2.90, *p*<0.0001) in line with the SEC-MALS data. The EC50 was also significantly higher at 245 ± 100 ng/mL compared to 19 ± 1 ng/mL for oligomerised Aβ42CC (the difference was 226 ng/mL; CI 95%, 136 to 316; *p*=0.0006). Incubation for an additional 16 h lead to even larger oligomers with 97% eluting as 1.6 MDa oligomers (see Additional Figure 5 in the Supplemental Appendix); however, the magnitude of the ALZ-201 ELISA response was maintained. The ATR-FTIR spectroscopy signal for these large oligomers was also indistinguishable from the signal for the 793-kDa oligomers (not shown).

To further define the binding profile of ALZ-201, its reactivity towards actively aggregating rAβ42 was investigated. Here, a ThT binding assay was used to assess the effect of ALZ-201 on fibril formation and sandwich ELISAs to detect ALZ-201-reactive oligomers formed during the aggregation. Standard curves for rAβ42CC oligomers were collected in parallel to estimate the number of ALZ-201-reactive oligomers present during aggregation.

**Fig. 2:**
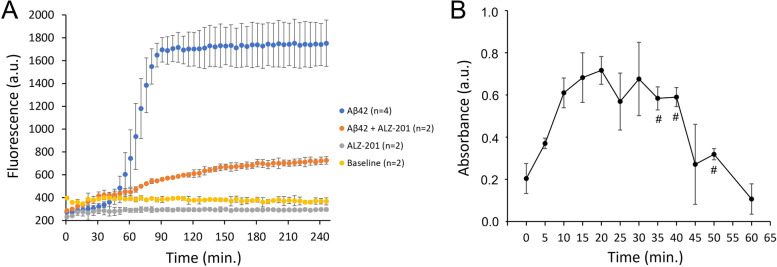
ALZ-201 effects on rAβ42 fibrillisation and reactive oligomers formed while actively aggregating rAβ42. **A** Thioflavin-T binding assay to assess the effect of ALZ-201 on fibrillisation of the rAβ42 peptide. **B** ALZ-201/ALZ-201 sandwich ELISAs detecting ALZ-201-reactive oligomers formed during actively aggregating rAβ42. # *n*=2; all other data points are *n*=3; the standard deviation (SD) = the errors

As expected based on the assay conditions used (physiological ionic strength, 37 °C, and shaking), rAβ42 underwent rapid and irreversible fibrillogenesis. Fibrils began accumulating after 45 min and the reaction was essentially complete after 90 min. Inclusion of ALZ-201 at 0.13 times the concentration of rAβ42 dramatically reduced, but did not completely inhibit fibril formation. The signal remaining in the presence of antibody indicated that unstructured monomeric rAβ42 could add directly to nucleated fibrils, thereby circumventing the oligomer state and supporting the view that oligomers are not obligate intermediates (Fig. 2A).

ALZ-201-reactive oligomers appeared instantly in the sandwich ELISAs, reaching a maximum after 20 min after which they slowly disappeared over 40 min as the rate of rAβ42 fibrillisation increased (Fig. 2B). Note that since the whole sample was used in this analysis, each data point contains the full spectrum of non-aggregated and aggregated Aβ42 species at each respective time point yet this had no impact on the antibody’s ability to distinguish the target from all these other forms. After 20 min, when the amount of rAβ42 oligomers reached maximum, only 39.4 ± 5.6 ng/mL of ALZ-201-reactive oligomers were present, corresponding to 0.047% of all Aβ42 (see Additional Figure 6 in the Supplemental Appendix). The more rapid rate of oligomerisation for rAβ42 in Fig. 2B, where a maximum can be observed at 20 min compared to Aβ42CC in Fig. 1B that shows only partial oligomerisation after 50 min, was anticipated as the experimental conditions (RT incubation versus shaking at 37°C) were different. Aggregation of the Aβ peptide is known to be both temperature dependent according to Arrhenius kinetics [74, 75] and influenced by agitation [76].

This demonstrates that ALZ-201 is reactive towards rAβ42 oligomer(s) and also suggests that while actively aggregating in vitro, only a minute fraction of the Aβ42 peptide is in this ALZ-201-reactive oligomeric state. Since the ALZ-201-positive signal disappeared (Fig. 2B) at the same time as the fibrils appeared (Fig. 2A), these rare oligomers ultimately seem to convert into fibrils either by a conformational switch into a cross-β structure or after first dissociating into monomeric peptides.

Thus, from these experiments, it can be concluded that ALZ-201 is stable and truly specific, not just selective, for non-fibrillar, soluble Aβ42CC and Aβ42 aggregates (i.e., structured oligomers) based on its unique conformational specificity for soluble aggregates of Aβ42CC and its ability to reduce rAβ42 fibrillisation and detect a low-abundant oligomeric species in actively aggregating rAβ42.

#### chALZ-201 retains its binding specificity towards Aβ42CC oligomers

To confirm the specificity of chALZ-201 and explore potentially unique binding characteristics of both ALZ-201 and chALZ-201 compared to existing mAbs, their binding specificity and affinity, as well as those of lecanemab, aducanumab and gantenerumab biosimilars, for monomeric and fibrillar Aβ42 and oligomeric Aβ42CC were evaluated using ELISA. Since this experiment was specifically designed to demonstrate the effect of different Aβ peptide conformations on antibody binding, ATR-FTIR analysis of the oligomeric Aβ42CC was used to confirm that these oligomeric aggregates do indeed have an anti-parallel β-sheet orientation in line with previous data [60] as opposed to the fibril structure for which the FTIR spectrum is indicative of a parallel orientation (Additional Figure 7 in the Supplemental Appendix). In addition, SEC-MALS demonstrated that the Mw of the oligomers in this experiment was 702 ± 4 kDa (see Additional Figure 2 in the Supplemental Appendix).

In the ELISA setup, the antigens were immobilised to the binding plates at high concentrations to reduce antigen multivalency effects. Any differences in binding would thus largely reflect conformational specificity or selectivity. Three major conformations of the Aβ42 peptide were studied here, namely the unstructured/random coil, anti-parallel β-sheet oligomers and parallel β-sheet fibrils represented by Aβ42 monomers coated at high pH, Aβ42CC oligomers (702 kDa), and Aβ42 fibrils, respectively.

**Fig. 3:**
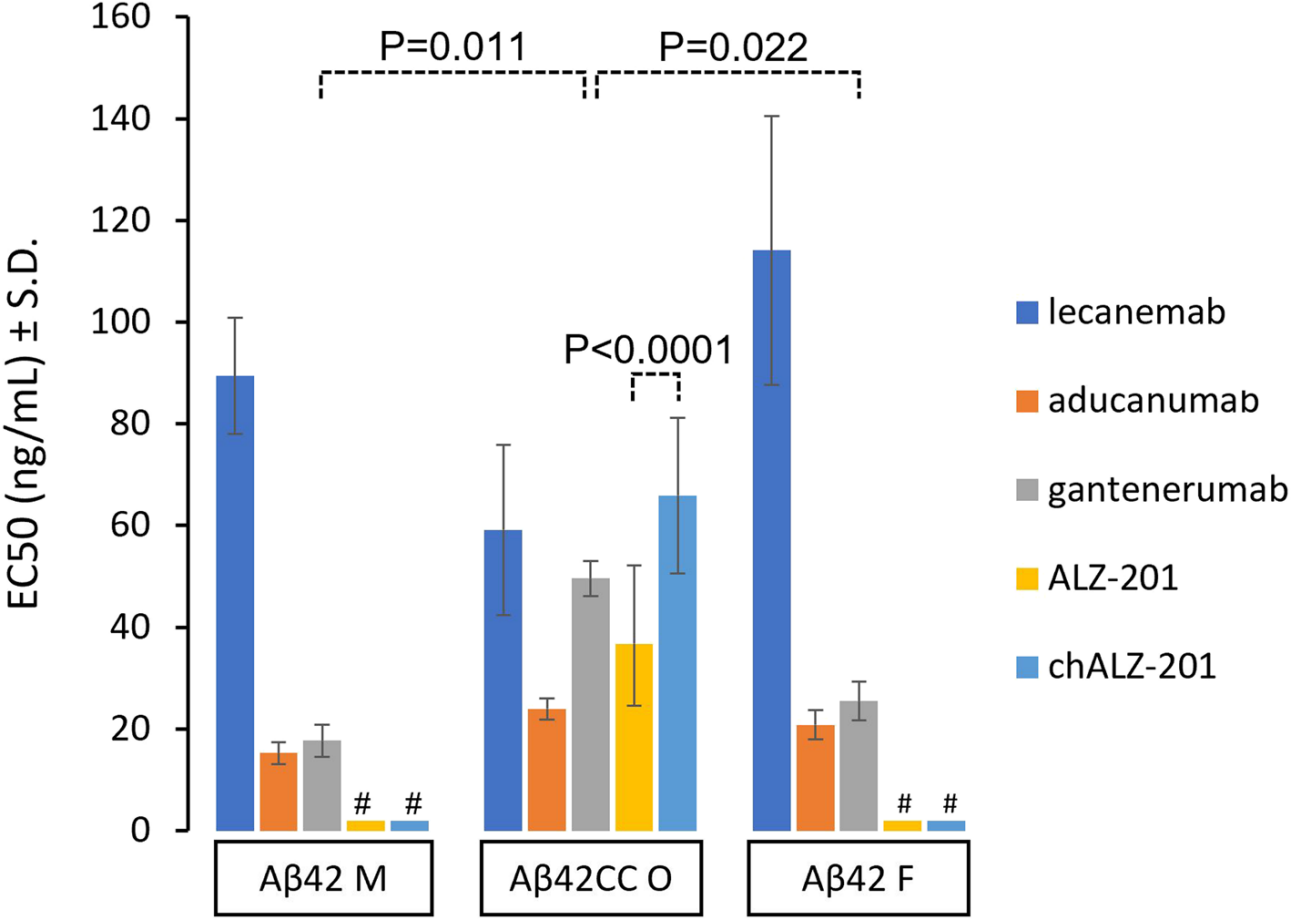
Half maximal effective concentration (EC50) of anti-Aβ antibody dose-response curves against three different Aβ42 forms. Antibody binding was measured using a direct ELISA against immobilised antigen, and the EC50 taken from a 4-parameter logistic equation fitted to the experimental data. Here, “M” denotes monomeric non-aggregated peptide, “O” oligomeric forms of the peptide with anti-parallel orientation of the β-sheets (see Additional Figure 7 in the Supplemental Appendix) and an oligomer content of 94% with an average Mw of 702 ± 4 kDa as determined by SEC-MALS (see Additional Figure 2 in the Supplemental Appendix), and “F” fibrillar forms of the peptide with a parallel orientation of the β-sheets (see Additional Figure 7 in the Supplemental Appendix) for which the Mw is unknown. All ELISAs were carried out in duplicates except for ALZ-201 and chALZ-201 against Aβ42CC O, for which *n*=68 and *n*=18, respectively. #: No binding detected. The error is the standard deviation (SD). See Additional Figure 8 in the Supplemental Appendix for experimental data

In line with the foregoing assays, no reactivity of ALZ-201 or chALZ-201 towards unstructured or fibrillar Aβ42 was detected. Murine ALZ-201 and chALZ-201 exhibited a small yet statistically significant difference in EC50 for the oligomeric form by 29 ng/mL (95% CI, 22.3267 to 35.9192, *p*<0.0001); however, they bound no other conformations of the peptide. In contrast, lecanemab, aducanumab, and gantenerumab biosimilars had similar affinities for the different peptide forms (*p*>0.05), with the possible exception of gantenerumab for which the EC50 against oligomers was 32 and 24 ng/mL higher compared to monomers (95% CI, 17.56 to 46.18, *p*=0.011) and fibrils (95% CI, 8.3893 to 39.8357, *p*=0.022), respectively (Fig. 3; see also Additional Figure 8 in the Supplemental Appendix).

Thus, the specific antigen binding properties to oligomeric Aβ42CC observed for ALZ-201 was retained in the chimeric human IgG1 variant chALZ-201. Neither antibody exhibited detectable reactivity towards unstructured monomeric Aβ42 or Aβ42 in fibril conformation. In contrast, the lecanemab, aducanumab and gantenerumab biosimilars (all of which target the N-terminal region of Aβ) did not significantly discriminate between these different Aβ structures, with the low *n* (*n*=2) precluding any clear conclusions for gantenerumab.

#### chALZ-201 does not bind plaques in human AD

**Fig. 4:**
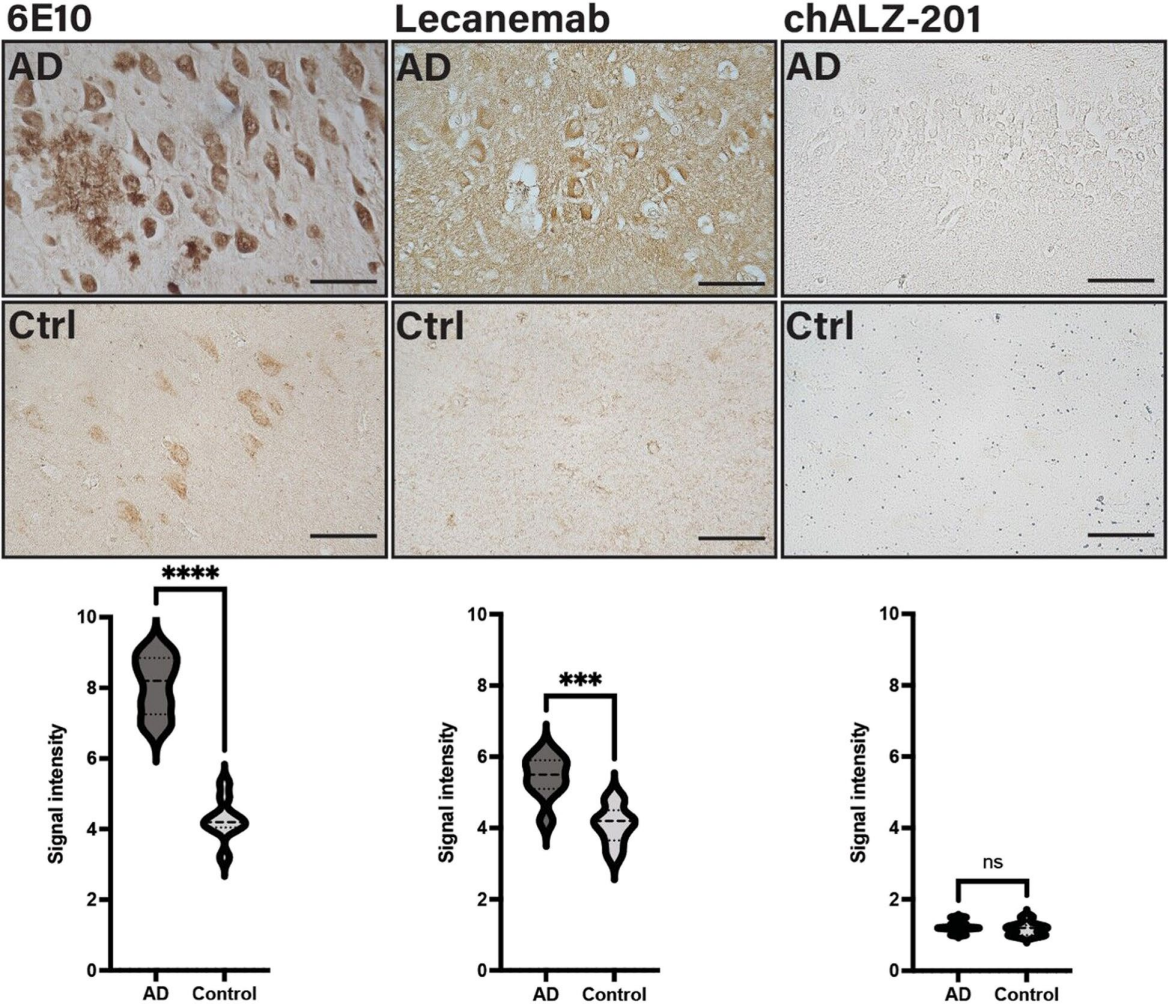
6E10, lecanemab biosimilar, and chALZ-201 immunohistochemistry in the hippocampus of human AD cases and controls. Immunohistochemistry was performed on the hippocampus of AD patients and controls using 6E10, a lecanemab biosimilar, and chALZ-201. Only 6E10 and the lecanemab biosimilar exhibited immunoreactivity towards the plaques, as was expected for antibodies that target the generic N-terminal of Aβ. In contrast, ALZ-201 did not bind to these deposits indicating that it is truly conformation specific and does not react with fibrillar Aβ. Graphs represent the difference in signal intensity between AD and controls. The black bar indicates a distance of 100 μm. ns=not significant, ****p≤0.0001, ***p≤0.001

To confirm that the non-reactivity of ALZ-201 and chALZ-201 towards fibrillar Aβ (yet strong reactivity towards AβOs) translates into human AD, the reactivity of chALZ-201 to fibrillar deposits, or plaques, in the hippocampus of subjects with AD was evaluated using IHC analysis. The binding of chALZ-201 to human AD tissue was compared with that of the lecanemab biosimilar, and 6E10 as a positive control. As expected, only lecanemab and 6E10 could be detected on the plaques since they strongly bind the N-terminal section of the Aβ peptide in plaques. In contrast, chALZ-201 did not bind to these fibrillar deposits in detectable amounts (Fig. 4).

The results indicate that chALZ-201 is a truly conformation-specific antibody in contrast to lecanemab and that the oligomer-specific epitope is not structurally preserved or retained during processing or is simply not present in sufficient amounts detectable with common IHC analysis.

### ALZ-201 reduces the neurotoxicity of human AD brain extracts isolated post mortem

Since ALZ-201 was found to specifically target a low-abundant Aβ42 oligomer, but not fibrils or monomers of the same peptide, it was posited that ALZ-201 could have therapeutic potential provided that the targeted oligomeric Aβ42 is a toxic species. Thus, a novel in vitro primary neuron model based on AD-patient-derived neurotoxic Aβ was used to investigate a potential neuroprotective effect of ALZ-201, as well as its effect on Aβ levels in human AD brain extracts to ascertain the potential neurotoxic Aβ species.

#### Brain extracts from post-mortem human AD tissue contain Aβ42 within the Aβ pool

Firstly, the spectrum of Aβ species and their relative levels in brain homogenate isolated and prepared from post-mortem brain tissue samples from individuals confirmed as pathological AD (*n*=7) or non-AD (*n*=3) (Table 1) prior to immunodepletion was determined. The analysis of Aβ content in whole-brain extracts from human brain tissue samples using Western blot, however, was insufficiently sensitive for quantification of the different fractions of Aβ (the estimated limit has been stated as being, typically, 1 ng/well [54]). Instead, Aβ levels were quantified using the more sensitive MSD Amyloid-β Peptide Panel 1 kit. As expected, there were higher levels of Aβ40 and Aβ42 peptides in AD brain samples based on pathology with variability between the different cases compared to controls, and much higher levels of Aβ40 than those of Aβ42 for both AD and control samples (Table 1). Thus, the human post-mortem AD brain tissue extracts contain the Aβ42 species at levels associated with AD pathology.

**Table 1:** Neuropathological classification of human brain tissue, demographics and protein content, including amyloid-β levels

Subject ID	Age	Gender	IHC		Protein content (mg/mL)	Aβ40 (pg/mg protein)	Aβ42 (pg/mg protein)
			Amyloid	p-Tau			
CO1	87	Male	−	−	1.43	29.27	2.88
CO2	60	Male	−	−	1.22	35.63	2.99
CO3	62	Female	−	−	0.77	47.80	6.15
AD1	85	Female	+	+	0.87	214.94	75.63
AD2	95	Male	+	+	1.43	67.30	43.63
AD3	72	Male	+	+	2.49	44.12	43.32
AD4	80	Female	+	+	0.49	248.25	74.77
AD5	96	Female	+	+	1.71	393.83	86.28
AD6	86	Female	+	+	1.05	220.54	273.75
AD7	60	Male	+	+	1.99	210.23	159.43

Pathological human cases of Alzheimer’s disease (AD, Braak stages 4–5; *n*=7) and non-AD controls (CO, *n*=3) were confirmed in tissue samples from the temporo-parietal grey matter cortices/frontal cortex that typically are affected by AD pathology using immunohistochemistry [77, 78]. Aβ40 and Aβ42 levels in homogenised whole-brain extracts from corresponding patients were measured by MSD assay and normalised to the total protein content as per the BCA assay.

#### The vast majority of Aβ in human AD brain extracts is not targeted by ALZ-201

**Fig. 5:**
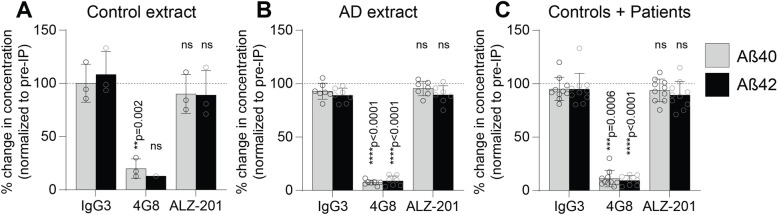
Effects of immunodepletion on amyloid-β (Aβ) levels in brain extracts. The percentage change of Aβ40 (grey) and Aβ42 (black) concentration to pre-immunodepleted (pre-IP) conditions (set to 100%) for **A** controls and **B** AD patients separately or **C** combined. Bar graphs show the mean ± SD, data points represent the individual values for control and/or AD patient brain extracts. Shapiro-Wilk normality testing was followed by parametric (ordinary) or non-parametric (Kruskall-Wallis) one-way ANOVA, and groups were compared to IgG3 control. Statistical testing confirmed that Aβ40/42 values did not differ between IgG3 control and pre-immunodepleted conditions. Note that Aβ42 levels were above the detection limit after 4G8 treatment for only one control case, resulting in a non-significant statistical difference due to an *n*=1 sample size. In the IgG3 condition in **C**, one outlier was detected and removed for subsequent analysis. Significance values are shown in the graph, ns = not significant

Since ALZ-201 was developed to uniquely target Aβ42 oligomers associated with AD pathology, the ability of ALZ-201 to deplete Aβ species found in the human brain extracts was then evaluated. ALZ-201, 4G8 (a positive control that depletes all Aβ) or IgG3 antibody (an isotype control to ALZ-201) were applied to AD and control brain extracts. Immunodepletion with 4G8 strongly depleted both Aβ42 and Aβ40 in brain extracts from control and AD cases, with Aβ42 levels below the detection limit (0.368 pg/mL as stated by the manufacturer) in two controls (CO1 and CO2) (Fig. 5). In contrast, neither ALZ-201 nor IgG3 significantly depleted the Aβ pool. These results confirm that ALZ-201 does not target the vast majority of Aβ present in human brain tissue.

#### Immunodepletion with ALZ-201 reduces neurotoxicity of brain extracts from post-mortem human AD tissue

Having demonstrated Aβ40 and Aβ42 species in human post-mortem brain extracts, the toxicity of human AD brain homogenate to primary mouse neurons in vitro using morphological parameters and the effect of ALZ-201 immunodepletion were assessed. This was in line with Hong et al. [54] who employed human induced pluripotent stem cell (iPSC)-derived neurons and validated this methodology to quantify neurotoxicity using primary neurons in vitro.

**Fig. 6:**
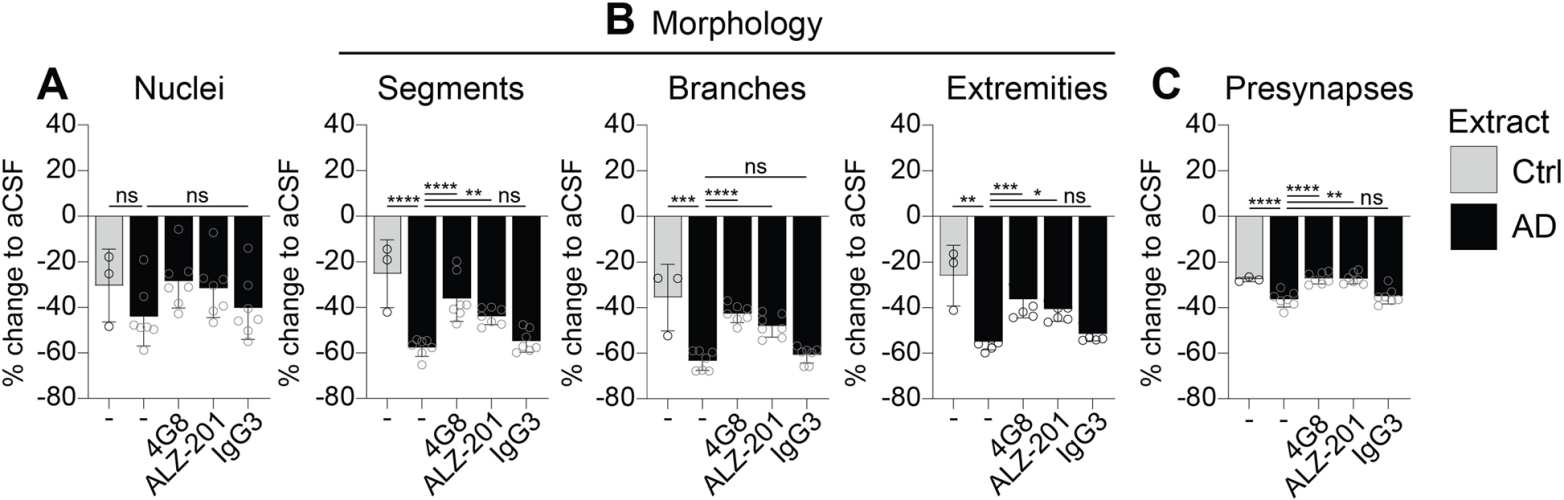
Effect of immunodepletion on the neurotoxicity of AD brain extracts. Primary mouse neurons were treated for 24 h with artificial cerebrospinal fluid (aCSF), extracts of non-AD controls (grey) or extracts of AD patients (black) that were either untreated (-) or immunodepleted with ALZ-201, 4G8 or IgG3. High-content automated microscopy was employed for the quantification of the number of **A** neuronal nuclei: **B** morphology (as determined by segments, branches and extremities), and **C** number of vGlut1-positive presynapses. Morphological and synaptic parameters were normalised against the number of neurons. Values are displayed as the % change to cultures treated with aCSF. Bar graphs show the mean ± SD and data points represent the individual values for control (grey) and (immunodepleted) AD brain extracts (black). Shapiro-Wilk normality testing was followed by parametric (ordinary) or non-parametric (Kruskall-Wallis) one-way ANOVA, and groups were compared to pre-immunodepleted conditions using Dunn’s or Dunnet’s post hoc test. Significance values: (**B**: segments) AD vs Ctrl <0.0001, vs 4G8 <0.0001, vs ALZ-201 =0.006; (**B**: branches) AD vs Ctrl = 0.0008, vs 4G8 <0.0001, vs ALZ-201 <0.0001; (**B**: extremities) AD vs Ctrl = 0.0053, vs 4G8 = 0.0004, vs ALZ-201 = 0.0145; (**C**: presynapses) AD vs Ctrl <0.0001, vs 4G8 <0.0001, vs ALZ-201 = 0.0011. ns = not significant

To this end, primary mouse neurons were treated for 24 h with aCSF, non-AD control extracts (*n*=3) or extracts from AD patients (*n*=7) that were either untreated or immunodepleted with ALZ-201, 4G8 or IgG3. High-content microscopy analysis was employed to quantify the number of neurons and synapses, as well as morphological complexity using dendrite (Map2) segments, branches and extremities, as a proxy for neurodegeneration. These data showed that compared to non-AD control, treatment with AD brain extracts did not lead to loss of neurons yet caused a significantly increased loss of dendrite segments and branches, as well as the number of presynaptic vGlut1 puncta per neuron (Fig. 6), showing neurotoxicity of AD brain extracts.

Importantly, immunodepletion of AD brain extracts with 4G8 significantly ameliorated the loss of dendrite segments and branches, as well as presynapses (Fig. 6), demonstrating a neuroprotective effect associated with the depletion of Aβ. Strikingly, immunodepletion with ALZ-201 significantly reduced the loss of these structures to a similar extent as 4G8 (Fig. 6). In contrast, and as expected, the negative control IgG3 had no effect on these structures, and immunodepleted control brains did not differ from the non-immunodepleted control brains in any of the assays (data not shown).

These results show that ALZ-201 and 4G8 can ameliorate AD neurotoxicity by binding a neurotoxic species. Unlike 4G8, however, which binds the total pool of Aβ as demonstrated by the MSD assay, it can be inferred that in line with its specific binding profile, ALZ-201 mediates this effect by binding Aβ42 oligomers that exist in very low yet toxic amounts in AD brains.

## Discussion

Here we report on the development and evaluation of the novel Aβ42-oligomer-specific mAb ALZ-201. We created ALZ-201 with the aim of being Aβ42-oligomer specific, which subsequent characterisation of its binding prolife confirmed. It proved to be specific for structured, oligomeric forms of the stabilised peptide Aβ42CC only (Fig. 1) and was able to prevent fibrillisation of Aβ42 (Fig. 2A). Yet ALZ-201 only reacted with very few species of non-physiologic Aβ42 (Fig. 2B), and binding to Aβ derived from AD patients’ brains was undetectable using standard research practices (Figs. 4 and 5). Notwithstanding, in a novel, in vitro primary neuron model based on AD-patient-derived neurotoxic Aβ, ALZ-201 effectively ameliorated neurotoxicity (Fig. 6).

The conformational ELISA assay described in our study (Fig. 3) demonstrated that while ALZ-201 specifically recognises an Aβ42CC oligomer with anti-parallel β-sheet content (determined by ATR-FTIR), it does not bind the Aβ42 fibril conformation dominated by parallel β-sheet structures or non-structured monomeric forms of the Aβ42 peptide. The smallest, stable rAβ42CC oligomer observed has been reported to be a spherical structure approximately 100 kDa in size and primarily with anti-parallel β-sheet structure content [60]. These oligomers were also shown not to convert to fibrils; instead, they form large, oligomeric structures with a protofibril-like appearance over time [60, 73]. Notably, the data in Fig. 1 demonstrate <30% binding of ALZ-201 to a sample of Aβ42CC, which SEC-MALS showed to be comprised of only 20% of oligomers approximately 100 kDa in size. Later Aβ42CC oligomerisation time points demonstrated an almost 100% presence of oligomers, albeit of different sizes (793 and 1600 kDa at 8 and 24 h of oligomerisation, respectively). Nevertheless, the ALZ-201 antibody dose-response curves were very similar irrespective of oligomer size (Additional Figure 4 in Supplementary Appendix). This is consistent with a binding model where ALZ-201 binds specifically to anti-parallel, β-sheet-rich oligomeric units approximately 100 kDa in size, and where binding strength is influenced by the presence of this Aβ conformation more than the size of the Aβ aggregates themselves.

The non-reactivity of ALZ-201 towards fibrils, including fibrillar Aβ in plaques of human AD brains, yet strong reactivity towards toxic Aβ42 oligomers is a very rare observation for an anti-Aβ antibody. This suggests that ALZ-201 may have superior therapeutic potential over other, non-specific, mAbs targeting Aβ in AD. While we were unable to unequivocally confirm the Aβ42 oligomer as the toxic species here, ALZ-201 efficiently neutralised the Aβ-mediated neurotoxicity in AD brain extracts to a similar extent as 4G8. This indicates that toxicity is indeed Aβ-dependent and driven by oligomeric forms of this peptide.

Interestingly, although the term “oligomer” is commonly used to collectively describe soluble toxic aggregates of Aβ, most of these oligomeric species appear to be non-toxic [54, 79]. Of note, Hong et al. [54] used experimental methodology similar to ours and demonstrated that the majority of soluble Aβ aggregates are indeed not bioactive and suggested that the levels of toxic soluble Aβ are too low to be detectable by Western blot analysis. This is in line with our results where, despite clear toxicity of AD patient samples, we could not detect the toxic species targeted by ALZ-201. Our results lead us to speculate that this particular “ALZ-201-reactive Aβ species” is a low-abundant, toxic oligomeric Aβ42 species that may largely account for Aβ toxicity in human AD.

The ability of ALZ-201 to effectively discriminate against the many different forms of the Aβ peptide in AD patients is likely to be of high clinical importance. The vast majority of systemically administered mAbs (typically only about 0.1% of mAbs cross the BBB by diffusion) will be subjected to a steady stream of non-toxic, peripheral Aβ42 and Aβ40 that, in AD patients, are present at 13.2 ± 7.2 and 244.3 ± 105.8 pg/mL, respectively [52] with half-life rates of approximately 3 h [53]. Furthermore, the levels of soluble Aβ are around 20-fold higher in the central nervous system (CNS) relative to peripheral levels [52] with approximately 3-fold longer half-lives [53]. Previously tested anti-Aβ mAbs, such as solanezumab and bapineuzumab, had high intrinsic affinities for Aβ peptides in general (both have *K*_D_ values in the low nM range [80]); therefore, they suffered from extensive target distraction and proved to be ineffective at reducing brain amyloid or affecting clinical outcomes [81, 82].

Similar negative clinical results have also been reported for crenezumab [83, 84], an anti-Aβ mAb developed using mouse hybridoma technology post immunisation with vaccine ACI-24 [85]. Crenezumab appears to have no conformational preference for any type of Aβ and binds Aβ monomers and oligomers with near-equal, low-nM affinity [85, 86]. In contrast, mAbs with higher selectivity for aggregated Aβ forms, including fibrillar and oligomeric Aβ (e.g. aducanumab [87], lecanemab [88] and gantenerumab [89]) or specificity for an Aβ species unique to plaques (e.g. donanemab that targets pyroglutamated Aβ [90]) will be less affected by non-aggregated peripheral and central Aβ; indeed, they have been shown to effectively reduce plaques in treated patients [91–94].

Notably, unlike conformation-specific ALZ-201, the reported selectivity for aggregated over non-aggregated Aβ forms for aducanumab, lecanemab and gantenerumab stems primarily from fast rates of complex dissociation. An antibody with a rapid dissociation rate (*k*_off_) will have a low affinity for individual antigens and less binding will occur if these are far apart, such as for Aβ in plasma and CSF. In contrast, the affinity will be much higher for structures with antigens in close proximity where rapid rebinding is more likely, as occurs with Aβ aggregates or Aβ monomers immobilised on ELISA plates. Indeed, the *k*_off_ values of the intrinsic affinity for aducanumab and gantenerumab are >1 s^−1^ and 1.5 × 10^−2^ s^−1^, respectively [80], and intermediate to these two values for lecanemab (0.16 s^−1^) [95]. These are high values―low-nM affinity antibodies typically have *k*_off_ rates in the order of <10^−5^ s^−1^. For these three mAbs, therefore, the size of the aggregate, and possibly also paratope bivalency to some extent [96], is a major determinant of functional affinity, not conformation, as our data strongly support (Fig. 3). Thus, these mAbs primarily will be targeting plaques with high functional affinity because these insoluble Aβ deposits, as well as being the largest multivalent Aβ structures in the brain, constitute >97% of all brain Aβ [31]. We note that gantenerumab was reported recently to only achieve a fraction of the expected effect on plaque reduction in two large phase 3 trials on AD patients [97]. This is in line with the hypothesis that high Aβ binding off-rates are a requirement for substantial plaque target engagement for antibodies specific for generic Aβ epitopes, where the 1.5 × 10^−2^ s^−1^ observed for gantenerumab could prove to have been insufficient.

Current clinical data suggest that the robust plaque removal observed for aducanumab, lecanemab, gantenerumab and donanemab is associated with a reduction in the rate of decline on composite scores of cognition and function for at least three of these mAbs [91–94]. Despite reaching statistical significance, the observed reductions in clinical decline associated with these therapies are, however, small, raising concerns as to whether the treatment effect is clinically meaningful or not [98]. Nevertheless, these therapies do reach the CNS in amounts sufficient for a pharmacological effect, indicating that while targeting and removing toxic Aβ species with immunotherapy in AD is a valid strategy, greater precision for oligomeric toxic species is likely needed.

Importantly, not only may the amount of plaque-targeting mAbs crossing the BBB to tackle central Aβ and induce clinically relevant effects be insufficient, but these mAbs are also associated with common, off-target side effects observed as ARIAs in MRI [47, 49, 57, 58]. ARIA pathophysiology is believed to be related to the targeting of both fibrillar parenchymal and vascular Aβ deposits leading to a loss of vessel integrity and increased leakage into brain tissue, a potential side-effect that will require MRI monitoring in clinical practice [99]. Stable mAbs with either higher selectivity or true specificity for oligomeric forms may, therefore, cross the BBB to engage with toxic Aβ in the brain more effectively and safely, and achieve significant clinical impact.

We note that lecanemab has been stated to also have higher functional affinity for Aβ protofibrils (large oligomers) than fibrils, which is counterintuitive based on the mechanism by which these N-terminal antibodies seem to achieve higher selectivity for aggregated Aβ (high *k*_off_). This claim appears to be based primarily on two peer-reviewed reports [100, 101], neither of which provide compelling data in support of such an effect since Aβ *size* was not defined as a control variable in either report. Sehlin et al. [101] even used fibril fragments obtained by sonication in an inhibition ELISA against immobilised protofibrils; this could significantly impact the results because sonication effectively disrupts fibrils into smaller pieces [102], thereby lowering lecanemab’s affinity for the fibrillar structures used in the experiment. Thus, it is impossible to draw any general conclusions regarding the relative affinity for AβO and Aβ fibrils using such methodology. The data we present in Fig. 3, and those presented on mAb158 by Englund et al. [88] (see Additional Figure 9 in the Supplemental Appendix), instead suggest that there are no meaningful effects of Aβ peptide conformation on lecanemab binding.

Although not studied here, we predict that other “oligomer-selective” anti-Aβ mAbs that target N-terminal Aβ sequences, such as ACU193 [103], will likely have similar binding profiles as lecanemab. Available peer-reviewed data do in fact suggest that ACU193 has a similar relative affinity for AβOs over monomers like lecanemab: both exhibit near-equal affinity for non-aggregated and aggregated Aβ in direct ELISAs [21] yet several hundred-fold differences in affinity for oligomers over monomers in competitive ELISAs (>200-fold for the lecanemab precursor mAb158 [88], and 650-fold for ACU193 [21]). Data for ACU193 reactivity against fibrils are not readily available; however, examples provided in filed patents suggest strong binding to fibrils. For example, an inhibition ELISA in U.S. Pat. No. 7,811,563 based on similar methodology used by Savage et al. [21] gave EC50 values of 10.0 ± 0.7 nM, 7.2 ± 0.6 nM and 104.7 ± 22 nM (errors are SE; *n* was not reported) for ACU193 mouse precursor 3B3 binding to AβO, fibrils and Aβ40, respectively. Unsurprisingly, 3B3 labelled plaques in samples from both transgenic APP/PS1 mice and human AD brains, which is to be expected for an anti-Aβ antibody that reacts with fibrils [104].

The results of our study thus indicate that ALZ-201 is a mAb with a unique, AβO-specific, binding profile compared to other mAbs claiming “oligomer selectivity”. Our findings may have major clinical implications and require further studies that will focus on determining which toxic species ALZ-201 targets in human AD brain and whether its neuroprotective effects in our in vitro model translate into other preclinical models of AD to improve cognitive dysfunction.

### Limitations

ALZ-201 was developed and its binding profile primarily characterised using an artificial disulphide-stabilised oligomer construct, Aβ42CC, which has been shown to resemble other similar artificial oligomers made from Aβ42 in in vitro settings [60, 73]. All such artificial oligomers are bound to be imitations of the true toxic oligomer present in the actual human condition. Although our work does not prove the relevance of the Aβ42CC oligomers to AD pathology, we found that an antibody specific for the conformational polymorph of these oligomers did in fact translate into human AD pathology by targeting toxic Aβ from AD patients. This finding supports continued research on Aβ42CC oligomers to further determine their relevance to the disease process.

In the ELISA experiment investigating the reactivity of ALZ-201 on actively aggregating rAβ42 (Fig. 2B), our objective was to determine if the oligomeric conformation observed in the 100-kDa Aβ42CC oligomer and the larger 700–1600 kDa protofibrils could be identified in this species of Aβ, and to what extent it was present. However, as we found that very few species were indeed ALZ-201-reactive, this precluded SEC-MALS analysis to determine if there was a preference for a particular size of Aβ42 oligomers. Such information could potentially elucidate the different species formed during the in vitro aggregation process of Aβ42, although this requires a different experimental setup and further studies. We believe, however, that data thus far collected on the ALZ-201 antibody strongly suggest that the oligomer conformation of the Aβ peptide is the main determinant of binding and that oligomer size is of lesser importance.

We chose to use whole-brain extracts instead of the soluble fraction to evaluate neurotoxicity and the potential impact of ALZ-201. Whole-brain extracts contain many different Aβ forms (soluble and insoluble), and we considered the potential of ALZ-201 to bind to the elusive target in this mixed pool of different forms of Aβ to be a rigorous assessment of its true specificity and consequent effects. Very little soluble Aβ42 exists in the brain yet virtually all Aβ toxicity observed in human-derived brain extracts stem from soluble Aβ42 oligomers [2, 79, 105] of which only a fraction appears to be toxic [54]. While we could not detect the specific toxic species in the pool targeted by ALZ-201, given its specific binding profile to synthetic Aβ, a low-abundant soluble species of Aβ42 is strongly implicated, supporting the findings of Hong et al. [54]. We employed mouse primary neurons, but observe similar AD brain homogenate-derived neurotoxicity as Hong et al. [54] who used a human iPSC-derived in vitro neuronal model. Our findings, therefore, are likely to translate to human iPSCs, and potentially to human neurons per se, which requires further study. We also acknowledge potential limitations of using biosimilars rather than the actual mAbs for the comparison of Aβ binding profiles with ALZ-201 specificity.

We recognise that evaluating ALZ-201 in a behavioural model of AD is of high interest. However, we find that relevant behavioural models for AD are limited; most animal models focus on Aβ plaque pathology as opposed to Aβ42-related oligomer pathology, and these do not translate into strong clinical efficacy [106]. Furthermore, other models may rely on artificial Aβ oligomers, but to truly evaluate the potential benefit of an anti-oligomer mAb, we believe, will require the use of human AD brain extracts in preclinical models. This is a challenging area that we are currently exploring, but that still requires complex methodology development and validation. These next-generation amyloid models are expected to provide a superior means to test the efficacy of ALZ-201 and other Aβ-targeting mAbs to rescue clinical phenotypes.

## Conclusions

This study confirms the binding specificity of ALZ-201, a unique mAb that binds to a conformational epitope on synthetic Aβ42CC oligomers and a subset of synthetic Aβ42 oligomers. Moreover, ALZ-201 depletes a toxic, non-fibrillar species in post-mortem AD brain extracts causing a positive physiological and protective impact on the integrity and morphology of mouse neurons. Based on the specificity of ALZ-201, we can infer that a subset of soluble, aggregated Aβ42 oligomers may contribute substantially to Aβ-mediated toxicity in AD patients, making them a viable target for drug development. This study supports further investigation of the potential effects of ALZ-201 in vivo as a disease-modifying candidate and a novel, highly selective Aβ42 oligomer-specific mAb for therapeutic intervention in AD.

## Supplementary Information

**Additional file 1:** **Figure 1.** SEC-MALS of an oligomeric recombinant Aβ42CC preparation.

**Additional file 2:** **Figure 2.** SEC-MALS of an oligomeric synthetic Aβ42CC preparation.

**Additional file 3:** **Figure 3.** Inhibition ELISA experiment.

**Additional file 4:** **Figure 4.** Antibody binding using a direct ELISA against different aggregated states of Aβ42CC.

**Additional file 5:** **Figure 5.** SEC-MALS of Aβ42CC at different oligomeric states.

**Additional file 6:** **Figure 6.** Standard curve for ELISA quantification of ALZ-201-reactive Aβ42 oligomers.

**Additional file 7:** **Figure 7.** ATR-FTIR on Aβ42 fibrils and Aβ42CC oligomers.

**Additional file 8:** **Figure 8.** One-site ELISAs against different Aβ species.

**Additional file 9:** **Figure 9.** Mab158 binding to protofibrils and fibrils.

## Data Availability

Data in raw format are available from the corresponding author, AS, upon reasonable request. The materials used herein, bar patient samples, are commercially available or can be custom ordered from commercial sources. Patient samples are bound by ethical restrictions and quantitative limits and cannot be made available.

## Abbreviations

aCSF: Artificial cerebrospinal fluid
AD: Alzheimer’s disease
ANOVA: One-way analysis of variance
APP: Amyloid precursor protein
ARIAs: Amyloid-related imaging abnormalities
ATR: Attenuated total reflection
Aβ: Amyloid-β
Aβ40: Aβ1–40, amyloid-β 1–40
Aβ42: Aβ1–42, amyloid-β 1–42
AβCC: Aβ with A21C/A30C mutations
AβO: Oligomeric amyloid-β aggregate/Aβ oligomer
BBB: Blood-brain barrier
BSA: Bovine serum albumin
chALZ-201: Chimeric ALZ-201
CI: Confidence interval
CNS: Central nervous system
CO: Non-AD control
CSF: Cerebrospinal fluid
DAB: 3,3'-Diaminobenzidine
DAPI: 4 ′,6-Diamidino-2-phenylindole
DIV: Day in vitro
dH_2_O: Distilled water
DMEM: Dulbecco’s modified Eagle’s medium
EC50: Half maximal effective concentration
ELISA: Enzyme-linked immunosorbent assay
FTIR: Fourier transform infrared
Hanks–HEPES: 4-(2-hydroxyethyl)-1-piperazineethanesulfonic acid
HI-FBS: Heat-inactivated foetal bovine serum
HPLC: High-performance liquid chromatography
IgG: Immunoglobulin G
IHC: Immunohistochemistry
iPSC: Induced pluripotent stem cell-derived neurons
*K*_D_: Antigen binding affinity
mAb: Monoclonal antibody
Map 2: Microtubule-associated protein 2
MRI: Magnetic resonance imaging
NGS: Normal Goat Serum
ns: Not significant
PBS: Phosphate buffer saline
PBS-T: Phosphate buffered saline containing 0.05% Tween20
PFA: Paraformaldehyde
pre-IP: Pre-immunodepleted
p-Tau: Phosphorylated tau
*P* value: Significance value
ROI: Region of interest
RT: Room temperature
SEC-MALS: Multi-angle light scattering coupled with size exclusion chromatography
SEC: Size exclusion chromatography
SD: Standard deviation
ThT: Thioflavin-T
vGlut1: Vesicular glutamate transporter 1
*Y*max: Maximum response
