# Supplementary material for: Aβ42 oligomer-specific antibody ALZ-201 reduces the neurotoxicity of Alzheimer’s disease brain extracts
Source: Alzheimers Res Ther. 2022 Dec 29;14:196. doi: 10.1186/s13195-022-01141-1 (PMC9798723; doi:10.1186/s13195-022-01141-1)
Supplement: Supplementary file 1 — Additional file 1: Figure 1. SEC-MALS of an oligomeric recombinant Aβ42CC preparation. [file 13195_2022_1141_MOESM1_ESM.docx]

**SUPPLEMENTARY APPENDIX**

**Additional Figure 1: SEC-MALS of an oligomeric recombinant Aβ42CC preparation**


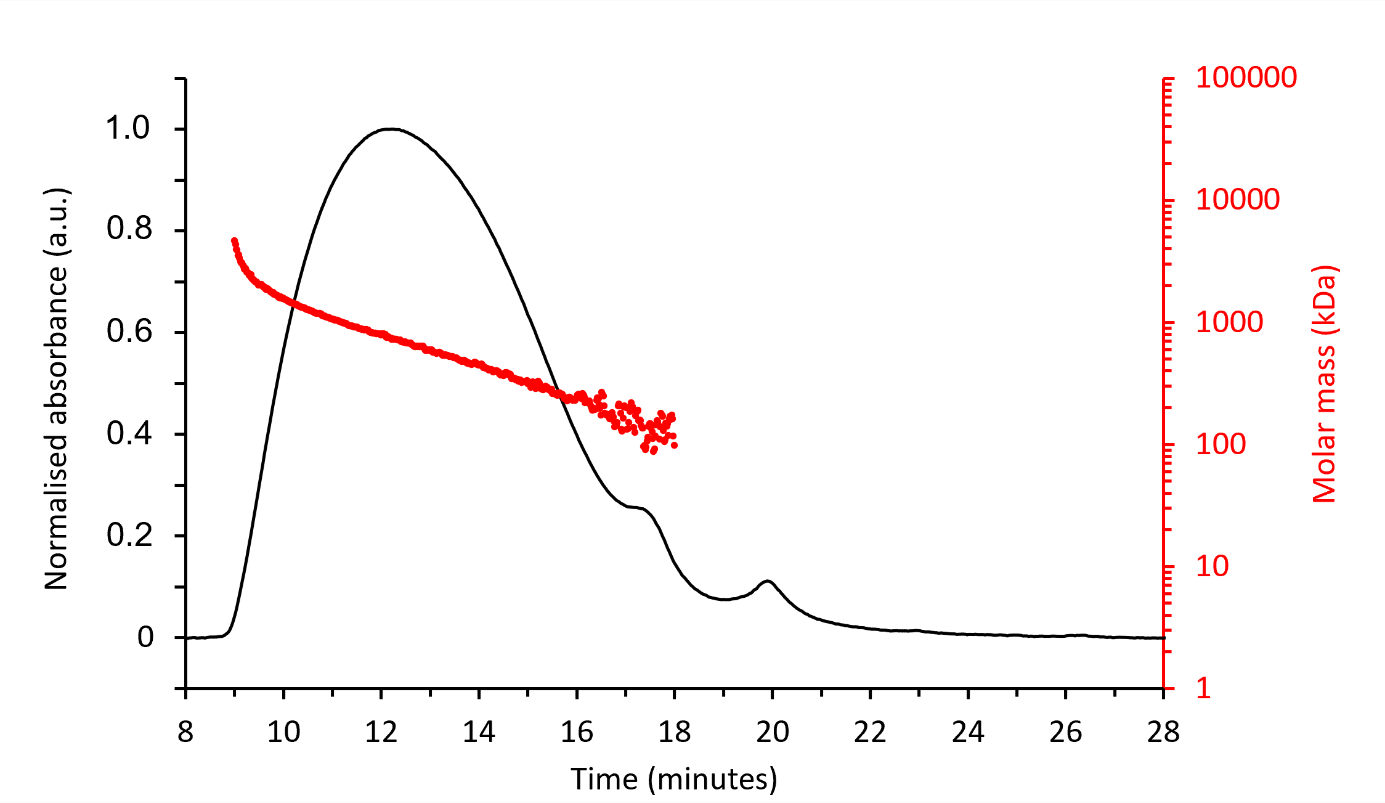


SEC-MALS of a 3.9 mg/mL oligomeric recombinant Aβ42CC preparation. UV detection at 280 nm demonstrated a broad peak eluting at 12.2 min (black curve) corresponding to a distribution of oligomers centred around 731 kDa as determined by MALS detection converted to molecular weight using ASTRA 6.1 Software (Wyatt Technology) (red data). The sample was analysed in duplicate, here only showing one trace for clarity. SEC-MALS=Multi-Angle Light Scattering coupled with Size Exclusion Chromatography.
