# Supplementary material for: Aβ42 oligomer-specific antibody ALZ-201 reduces the neurotoxicity of Alzheimer’s disease brain extracts
Source: Alzheimers Res Ther. 2022 Dec 29;14:196. doi: 10.1186/s13195-022-01141-1 (PMC9798723; doi:10.1186/s13195-022-01141-1)
Supplement: Supplementary file 2 — Additional file 2: Figure 2. SEC-MALS of an oligomeric synthetic Aβ42CC preparation. [file 13195_2022_1141_MOESM2_ESM.docx]

**Additional Figure 2: SEC-MALS of an oligomeric synthetic Aβ42CC preparation**

**
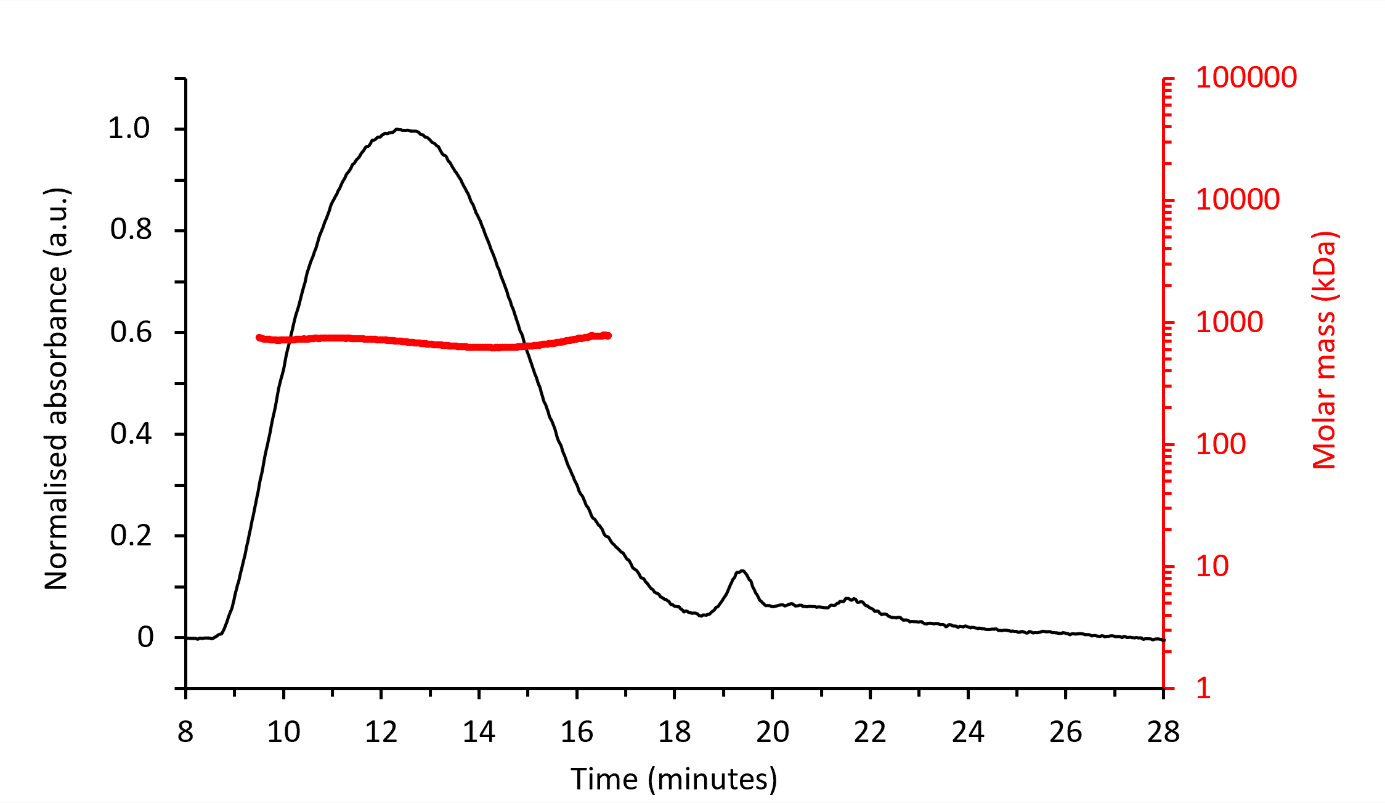
**

SEC-MALS of a 0.6 mg/mL oligomeric synthetic Aβ42CC preparation UV detection at 280 nm demonstrated a broad peak eluting at 12.5 min (black curve) corresponding to a distribution of oligomers centred around 702 ± 3.5 kDa, with an oligomer content of >94%, as determined by MALS detection converted to molecular weight using ASTRA 6.1 Software (Wyatt Technology) (red data). N=4 samples were analysed, here only showing one trace for clarity. SEC-MALS=Multi-Angle Light Scattering coupled with Size Exclusion Chromatography.
