# Supplementary material for: Aβ42 oligomer-specific antibody ALZ-201 reduces the neurotoxicity of Alzheimer’s disease brain extracts
Source: Alzheimers Res Ther. 2022 Dec 29;14:196. doi: 10.1186/s13195-022-01141-1 (PMC9798723; doi:10.1186/s13195-022-01141-1)
Supplement: Supplementary file 3 — Additional file 3: Figure 3. Inhibition ELISA experiment. [file 13195_2022_1141_MOESM3_ESM.docx]

**Additional Figure 3: Inhibition ELISA experiment**


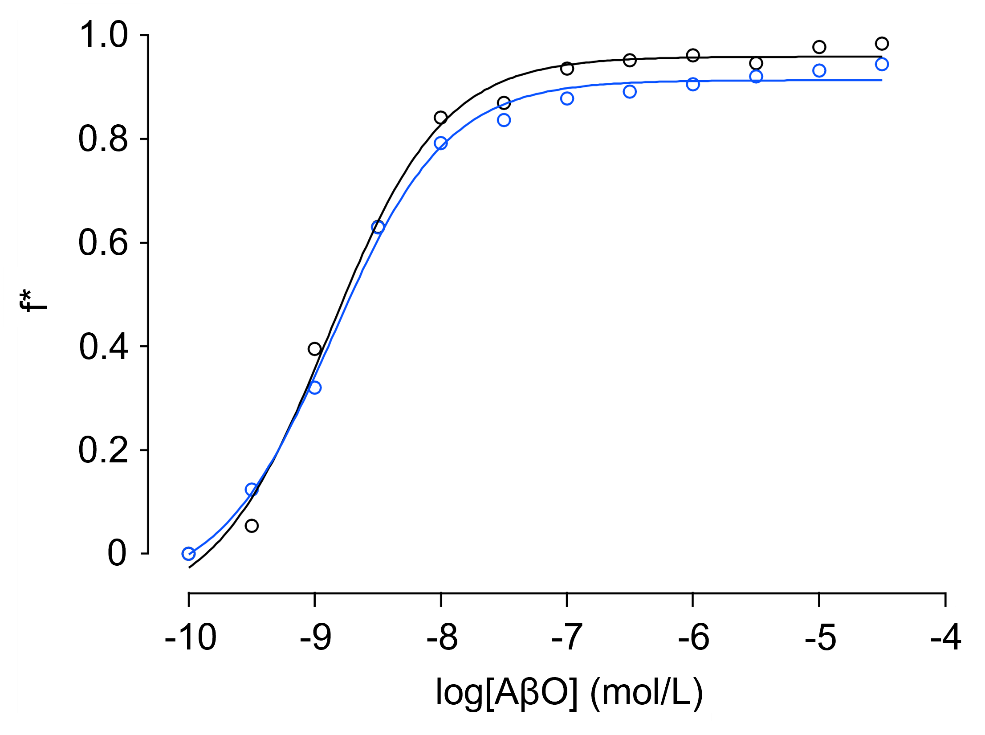


Inhibition ELISA experiment of two consecutive plates showing the first analysis (black curve) and the second analysis (blue curve). The dissociation constant (K_D_) for the antibody’s affinity for Aβ oligomers (AβOs) was taken as the concentration of antigen required to inhibit half of the ELISA signal by fitting a sigmoidal equation to the data using the programme IGOR (Wavemetrics). Here, *f** is the square root of the fraction of saturated antibody. The similarity of the two curves indicates that the equilibrium between antibody and antigen has not been disturbed during the first pre-incubation step. Two similar experiments gave an average K_D_ of 1.34 ± 0.29 nM. The error is the standard deviation (SD) of the curve-fitting procedure. ELISA=enzyme-linked immunosorbent assay.
