# Supplementary material for: Aβ42 oligomer-specific antibody ALZ-201 reduces the neurotoxicity of Alzheimer’s disease brain extracts
Source: Alzheimers Res Ther. 2022 Dec 29;14:196. doi: 10.1186/s13195-022-01141-1 (PMC9798723; doi:10.1186/s13195-022-01141-1)
Supplement: Supplementary file 4 — Additional file 4: Figure 4. Antibody binding using a direct ELISA against different aggregated states of Aβ42CC. [file 13195_2022_1141_MOESM4_ESM.docx]

**Additional Figure 4: Antibody binding using a direct ELISA against different aggregated states of Aβ42CC**

**
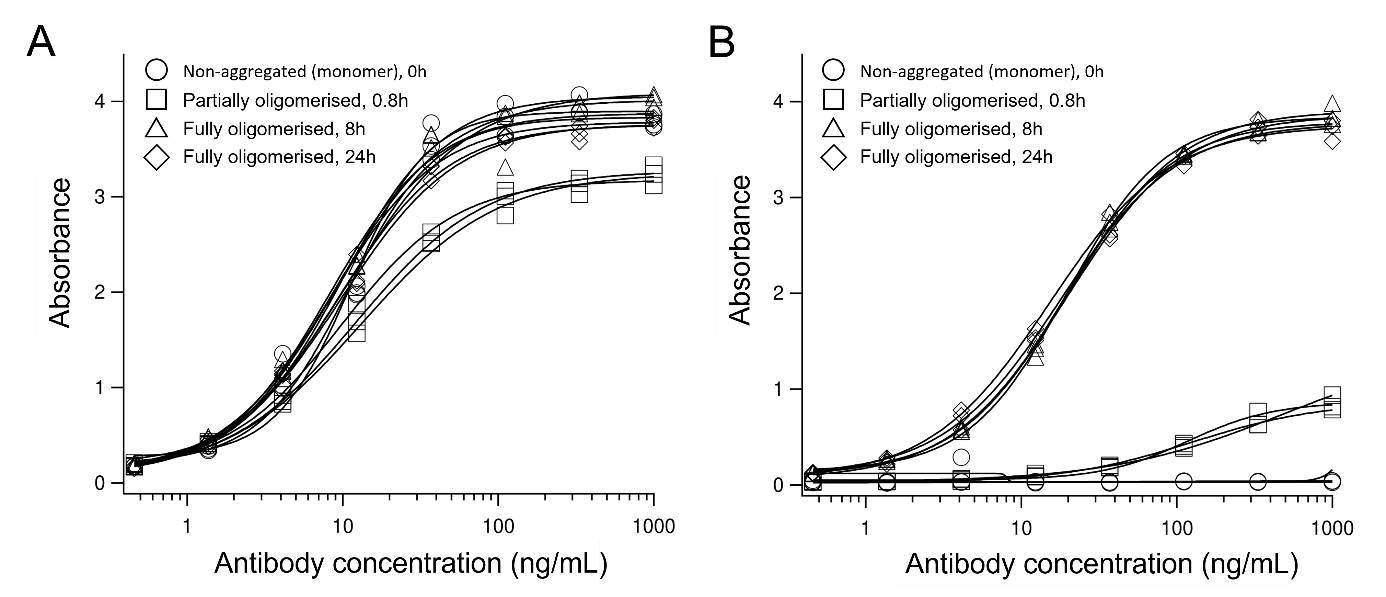
**

Data supporting Figure 1. Antibody binding was measured using a direct ELISA against different aggregated states of Aβ42CC: non-aggregated peptide at pH 10 (circles), partially oligomerised peptide after 50 min in PBS at pH 7.2 (squares), fully oligomerised peptide (793 kDa) after 8 h in PBS at pH 7.2 (triangles), and after 24 h (1600 kDa) in PBS at pH 7.2 (diamonds). Panel (A) shows data for the control antibody 6E10 that is specific for Aβ in general. Panel (B) shows data for ALZ-201, which is specific for a structure formed when Aβ42CC assembles into soluble aggregates. The solid lines are a 4-parameter logistic equation fitted to the experimental data from which the half maximal effective concentration (EC50) and maximum amplitude (Ymax) was extracted. ALZ-201 exhibits clear conformational specificity for structured oligomeric forms of the peptide with no binding to unstructured peptide (coated at pH 10) and only partial binding to incompletely oligomerised peptide (after 50 min of aggregation). All assays were carried out in triplicates. ELISA=enzyme-linked immunosorbent assay.
