# Supplementary material for: Aβ42 oligomer-specific antibody ALZ-201 reduces the neurotoxicity of Alzheimer’s disease brain extracts
Source: Alzheimers Res Ther. 2022 Dec 29;14:196. doi: 10.1186/s13195-022-01141-1 (PMC9798723; doi:10.1186/s13195-022-01141-1)
Supplement: Supplementary file 5 — Additional file 5: Figure 5. SEC-MALS of Aβ42CC at different oligomeric states. [file 13195_2022_1141_MOESM5_ESM.docx]

**Additional Figure 5: SEC-MALS of Aβ42CC at different oligomeric states**


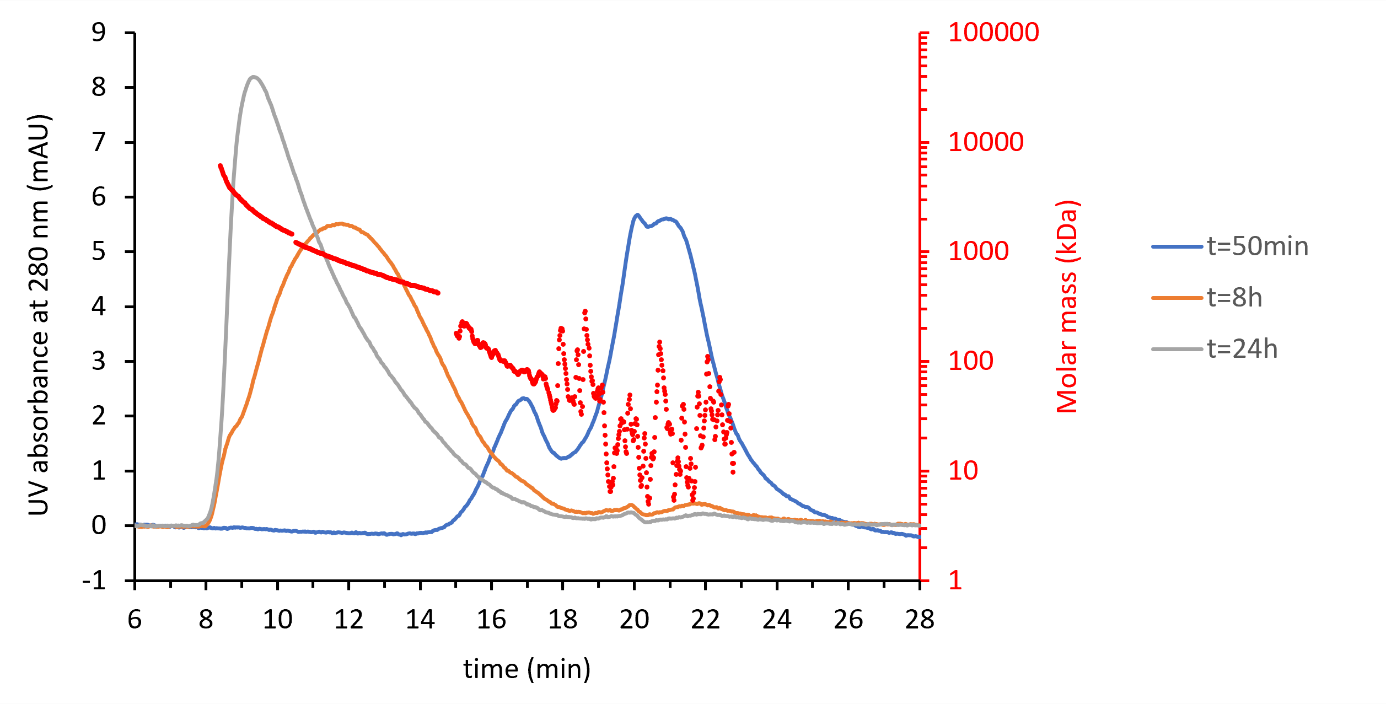


SEC-MALS of 0.6 mg/mL Aβ42CC at different oligomeric states obtained after incubation for 50 min, 8 h, and 24 h at 21 °C. UV detection at 280 nm demonstrates partially oligomerised peptide after 50 min with 19.5% of the peptide forming 104 kDa oligomers eluting at around 17 min and the rest as non-aggregated peptide at around 21 min. After 8 h incubation, fully oligomerised peptide was obtained after 12 min with a size distribution centred around 793 kDa. Longer incubation times lead to even larger aggregates eluting at or near the void volume of the column at 9 min. Samples allowed to oligomerize for 24 h have a molecular mass of approximately 1.6 MDa. The molecular mass was determined by MALS detection converted to molecular weight using ASTRA 6.1 Software (Wyatt Technology) (red data). Samples were analysed in duplicates. SEC-MALS=Multi-Angle Light Scattering coupled with Size Exclusion Chromatography.
