# Supplementary material for: Aβ42 oligomer-specific antibody ALZ-201 reduces the neurotoxicity of Alzheimer’s disease brain extracts
Source: Alzheimers Res Ther. 2022 Dec 29;14:196. doi: 10.1186/s13195-022-01141-1 (PMC9798723; doi:10.1186/s13195-022-01141-1)
Supplement: Supplementary file 6 — Additional file 6: Figure 6. Standard curve for ELISA quantification of ALZ-201-reactive Aβ42 oligomers. [file 13195_2022_1141_MOESM6_ESM.docx]

**Additional Figure 6: Standard curve for ELISA quantification of ALZ-201-reactive Aβ42 oligomers**


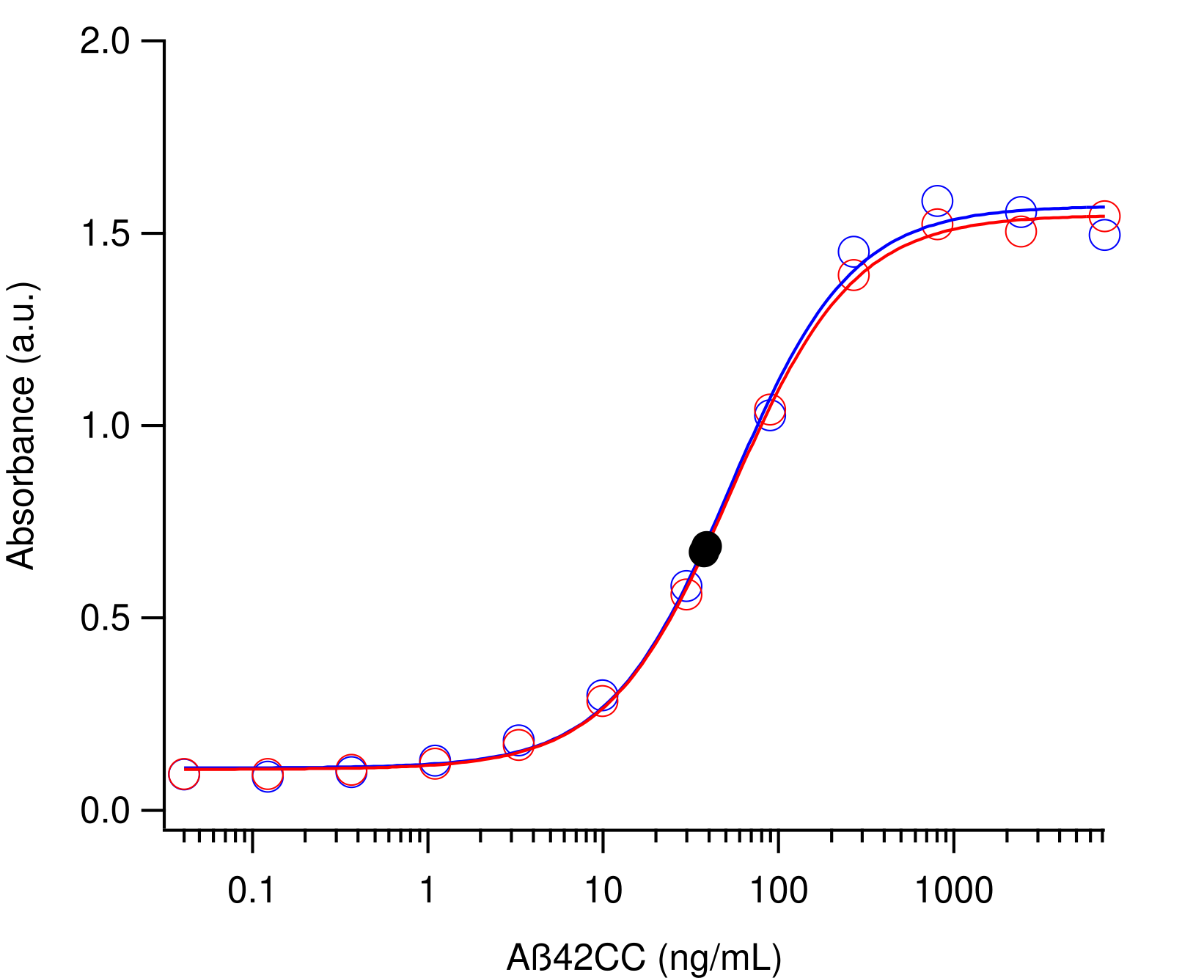


An example of a standard curve (n=2) run in parallel during the quantification of ALZ-201-reactive Aβ42 oligomers formed during actively aggregating recombinant Aβ42 using ELISA. Here, stable recombinant Aβ42CC oligomers were used as reference. Two series were collected, blue and red. These were very similar. The two black circles represent data obtained for Aβ42 at 20 min of aggregation, for which the standard curve indicates that the absorbance measured corresponds to 43.4 and 41.8 ng/mL ALZ-201-reactive Aβ42 oligomers. The solid lines are the fits of a 4-parameter logistic function to the data. ELISA=enzyme-linked immunosorbent assay.
