# Supplementary material for: Aβ42 oligomer-specific antibody ALZ-201 reduces the neurotoxicity of Alzheimer’s disease brain extracts
Source: Alzheimers Res Ther. 2022 Dec 29;14:196. doi: 10.1186/s13195-022-01141-1 (PMC9798723; doi:10.1186/s13195-022-01141-1)
Supplement: Supplementary file 7 — Additional file 7: Figure 7. ATR-FTIR on Aβ42 fibrils and Aβ42CC oligomers. [file 13195_2022_1141_MOESM7_ESM.docx]

**Additional Figure 7: ATR-FTIR on Aβ42 fibrils and Aβ42CC oligomers**


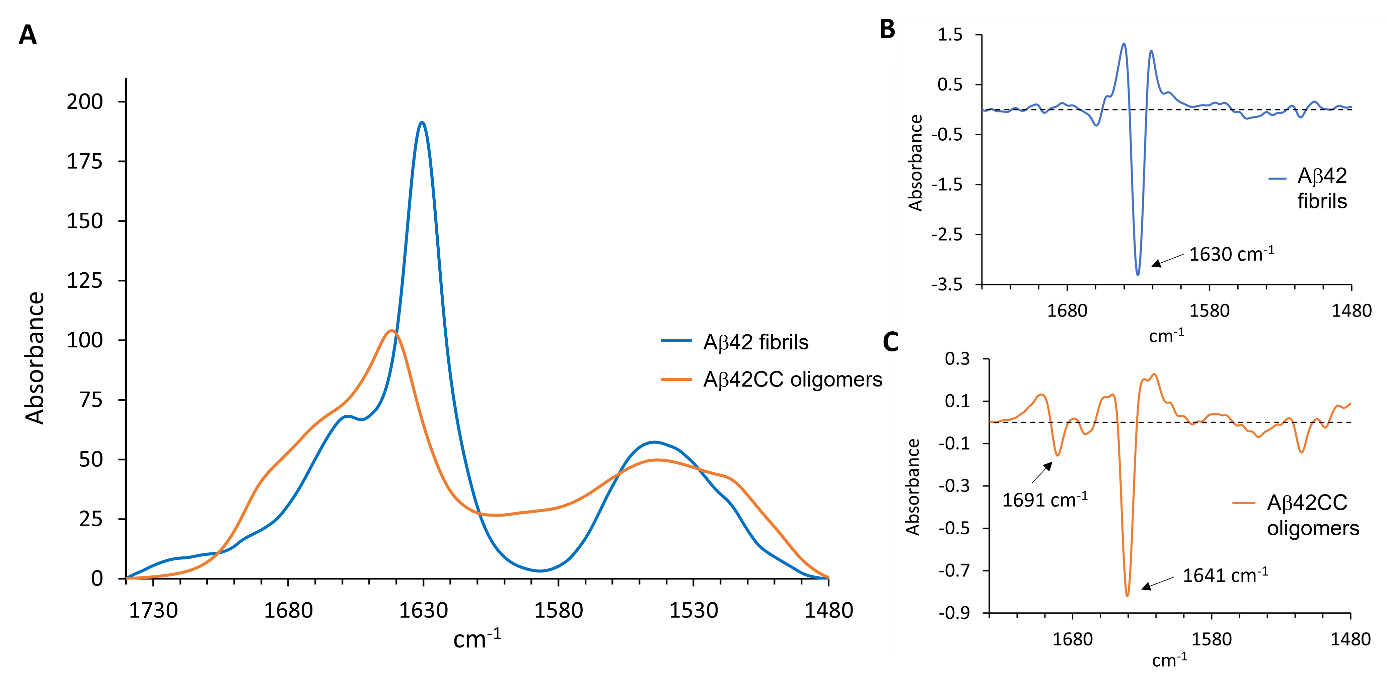


Comparison of (A) the mean Fourier transform infrared (FTIR) spectra recorded for Aβ42 fibrils and 702-kDa Aβ42CC oligomers (protofibrils); (B) the second derivative of the mean spectrum for Aβ42 fibrils; and, (C) the second derivative of the mean spectrum for Aβ42CC oligomers (protofibrils). Second derivatives were calculated using 9-points Savitzky-Golay smoothing. The spectral region related to protein absorption is shown (1740-1478 cm^-1^). As stated in the literature cited in the main manuscript, anti-parallel β-sheet structures in oligomers display two stretching frequency absorptions in the Amide I region: one major at ~1630 cm^-1^ and one in the order of five-fold weaker at around 1695 cm^-1^. For the parallel β-sheet structures in fibrils, however, only the major component is expected. The data for Aβ42CC oligomers thus demonstrate that these are composed of anti-parallel β-sheet structures. An estimation of the secondary structure content of Aβ42CC oligomers, based on a database of FTIR spectra of proteins available at Spectralys Biotech (Brussels, Belgium), demonstrated 0.0% α helix, 39.8±0.7% β-sheet, 16.5±0.2% turn, and 41.1±0.2% coil content.
