# Supplementary material for: Aβ42 oligomer-specific antibody ALZ-201 reduces the neurotoxicity of Alzheimer’s disease brain extracts
Source: Alzheimers Res Ther. 2022 Dec 29;14:196. doi: 10.1186/s13195-022-01141-1 (PMC9798723; doi:10.1186/s13195-022-01141-1)
Supplement: Supplementary file 8 — Additional file 8: Figure 8. One-site ELISAs against different Aβ species. [file 13195_2022_1141_MOESM8_ESM.docx]

**Additional Figure 8: One-site ELISAs against different Aβ species**


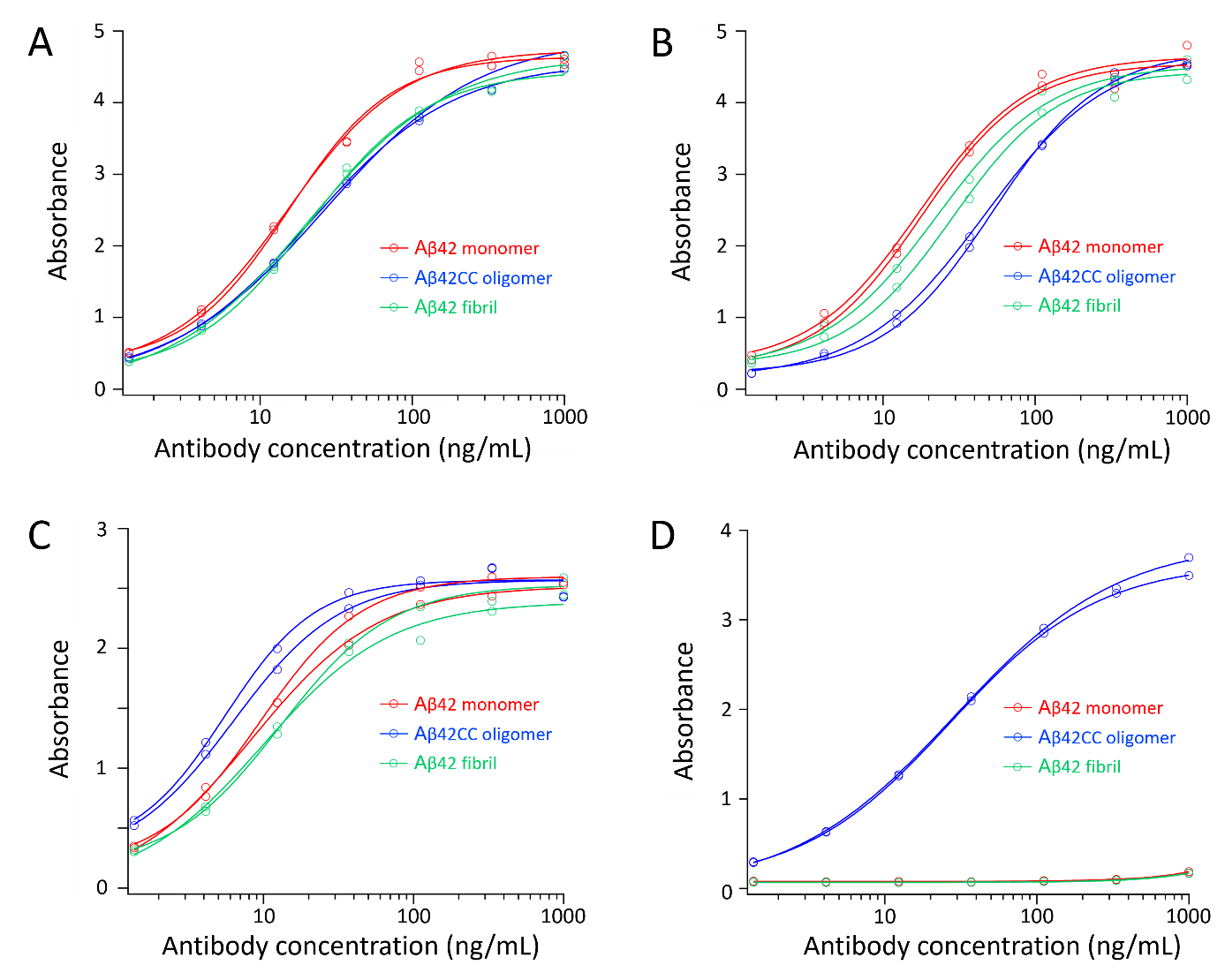


Data supporting Figure 3. One-site ELISAs against monomeric Aβ42 (red), oligomeric Aβ42CC (702 kDa; blue), and fibrillar Aβ42 (green) for (A) aducanumab biosimilar, (B) gantenerumab biosimilar, (C) lecanemab biosimilar, and (D) chALZ-201 (two representative experiments are shown). Only chALZ-201 exhibits specificity for the oligomeric conformation, whereas aducanumab, gantenerumab, and lecanemab are specific for Aβ and, hence, do not exhibit any meaningful discrimination potential for different conformations of the Aβ peptide. The solid lines are the fits of a 4-parameter logistic function to the data. ELISA=enzyme-linked immunosorbent assay.
