# Supplementary material for: Aβ42 oligomer-specific antibody ALZ-201 reduces the neurotoxicity of Alzheimer’s disease brain extracts
Source: Alzheimers Res Ther. 2022 Dec 29;14:196. doi: 10.1186/s13195-022-01141-1 (PMC9798723; doi:10.1186/s13195-022-01141-1)
Supplement: Supplementary file 9 — Additional file 9: Figure 9. Mab158 binding to protofibrils and fibrils. [file 13195_2022_1141_MOESM9_ESM.docx]

**Additional Figure 9: Mab158 binding to protofibrils and fibrils**


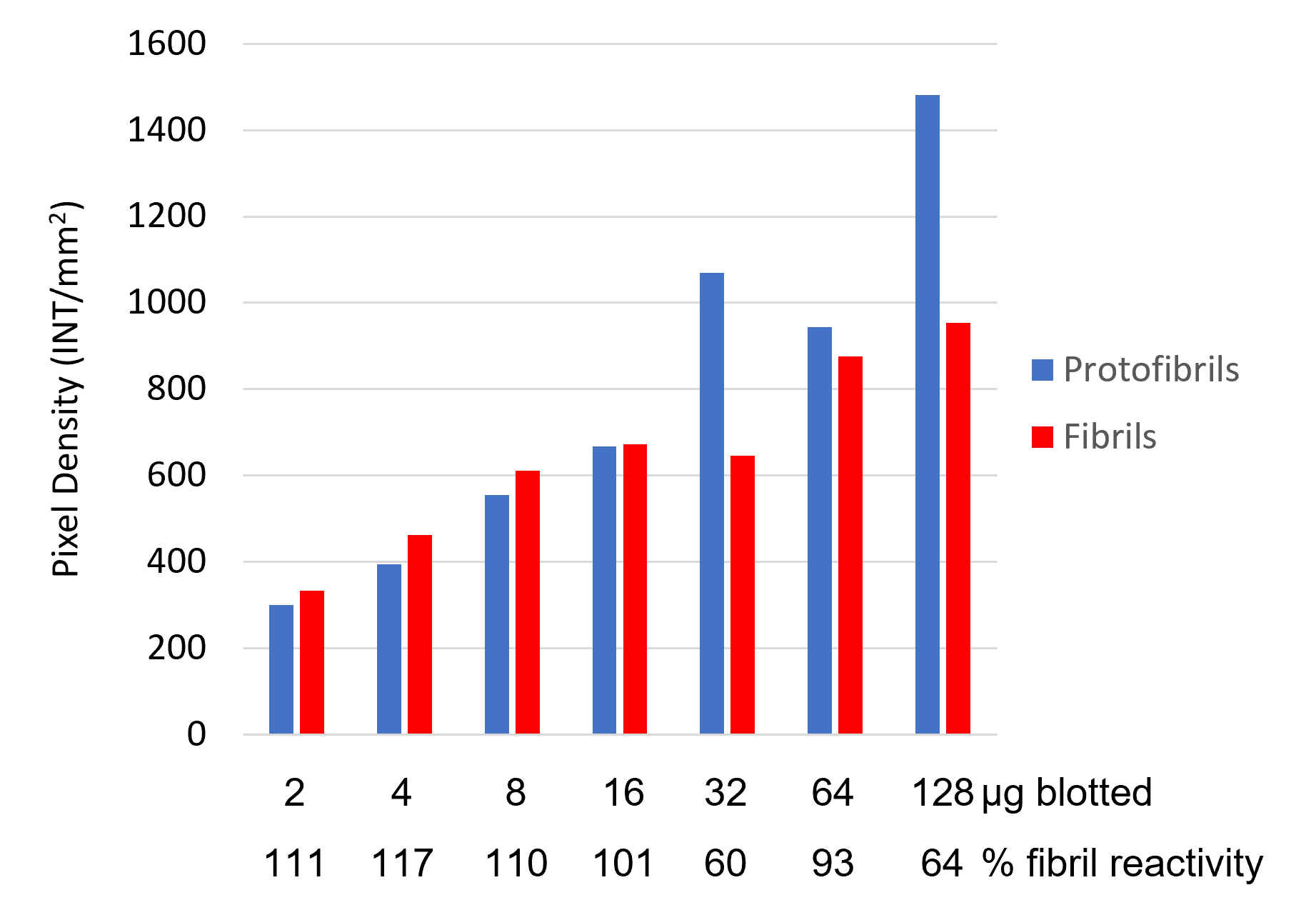


Englund *et al*. [1] prepared protofibrils by reconstituting lyophilized synthetic Aβ42 (PolyPeptide Laboratories, Germany) in 10 mM NaOH, diluting it to 443 µM in 10 × PBS, and incubating overnight at 37 °C. Here, protofibrils were defined as soluble oligomers ≥100 kDa eluting in the void on a Superdex 75 size-exclusion chromatography column (GE-Amersham, Uppsala, Sweden). Fibrils were prepared by reconstituting lyophilized recombinant peptide (rPeptide, USA) in 10 mM NaOH, diluting it to 50 µM in 2 x PBS, and incubating it >48 h. Protofibrils were diluted to 50 µM, and both fibrils and protofibrils were blotted onto a nitrocellulose membrane (BioRad, USA) and reactivity against mAb158 assayed using standard experimental procedures (see Englund *et al*. [1] for details). The data, presented in Figure 3A in Englund *et al*. [1], were imported here for quantification. An identically sized area was thus defined around each blot and pixel density measured and plotted. Results indicate that the reactivity against fibrils was 94 ± 23% that of protofibrils (the error is the standard deviation, SD) so there was no difference in the amount of N-terminal binding sites on fibrils and protofibrils of Aβ42, suggesting that mAb158 seems to bind both forms equally well. [1] Englund H, Sehlin D, Johansson AS, Nilsson LN, Gellerfors P, Paulie S, *et al*. Sensitive ELISA detection of amyloid-beta protofibrils in biological samples. J Neurochem. 2007;103(1):334-45.
